# Validating an algebraic approach to characterizing resonator networks

**DOI:** 10.1038/s41598-023-50089-1

**Published:** 2024-01-15

**Authors:** Viva R. Horowitz, Brittany Carter, Uriel F. Hernandez, Trevor Scheuing, Benjamín J. Alemán

**Affiliations:** 1 Physics Department, Hamilton College, Clinton, NY 13323 USA; 2 Department of Physics, University of Oregon, Eugene, OR 97403 USA; 3 Materials Science Institute, University of Oregon, Eugene, OR 97403 USA; 4 Center for Optical, Molecular, and Quantum Science, University of Oregon, Eugene, OR 97403 USA; 5 Phil and Penny Knight Campus for Accelerating Scientific Impact, University of Oregon, Eugene, OR 97403 USA

**Keywords:** Physics, Applied mathematics

## Abstract

Resonator networks are ubiquitous in natural and engineered systems, such as solid-state materials, electrical circuits, quantum processors, and even neural tissue. To understand and manipulate these networks it is essential to characterize their building blocks, which include the mechanical analogs of mass, elasticity, damping, and coupling of each resonator element. While these mechanical parameters are typically obtained from response spectra using least-squares fitting, this approach requires a priori knowledge of all parameters and is susceptible to large error due to convergence to local minima. Here we validate an alternative algebraic means to characterize resonator networks with no or minimal a priori knowledge. Our approach recasts the equations of motion of the network into a linear homogeneous algebraic equation and solves the equation with a set of discrete measured network response vectors. For validation, we employ our approach on noisy simulated data from a single resonator and a coupled resonator pair, and we characterize the accuracy of the recovered parameters using high-dimension factorial simulations. Generally, we find that the error is inversely proportional to the signal-to-noise ratio, that measurements at two frequencies are sufficient to recover all parameters, and that sampling near the resonant peaks is optimal. Our simple, powerful tool will enable future efforts to ascertain network properties and control resonator networks in diverse physical domains.

## Introduction

**Figure 1:**
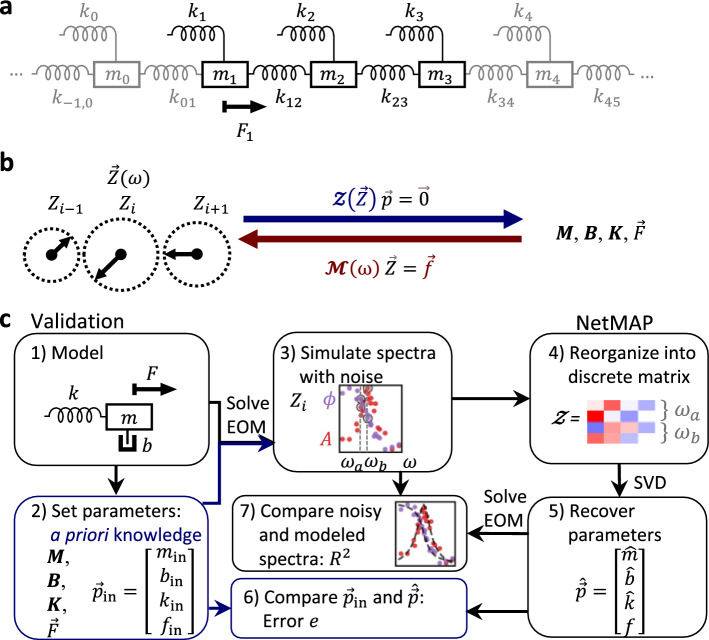
(**a**) A resonator network as a chain of mass and spring resonators. (**b**) We calculate the relationship between the complex amplitude $\vec{Z}(\omega)$ of the masses and the underlying parameters. NetMAP (top blue arrow) solves the inverse problem to calculating spectra (red arrow). (**c**) Steps of the validation process: (1) Modeling a mass-spring network, such as a monomer, shown here, or dimer. (2) Setting the parameters $\vec{p}_\text{in}$ to simulate. (3) Solving the equations of motion (EOM) and simulating spectra with noise. (4) Using simulated measurements of amplitude and phase at discrete frequency points to construct a matrix. (5) Using Singular Value Decomposition (SVD) to recover the parameters $\widehat{\vec{p}}$, and scaling the result as needed. Here $f$ is shown without a hat to identify it as a known quantity while the others are scaled to $f$. (6) Calculating the percent error $e$ to compare the recovered parameters to the set parameters. (7) Calculating the expected spectra for the recovered parameters, and calculating ${R}^{2}$ to compare the simulated data and the expected curve. We find that ${R}^{2}$ is correlated with error $e$.

Resonator networks are a ubiquitous^1^ and diverse class of systems, found in both natural^2^ and engineered contexts. They can range in scale from astronomical systems^3^ to biological and neural networks^4–6^, and from small-scale micromechanical lattices^7,8^ to large integrated circuits^9^ and solid-state materials. These many-body systems exhibit rich collective behaviors, such as brain memory, quantum^10,11^ and classical computation, and optical properties of solids, making them an important subject of study. To understand and engineer this behavior, it is necessary to characterize the network building blocks^7^ (e.g., neurons, micromechanical resonators, ions, etc.) and their connectivity. For example, quantum processors and tunable mechanical metamaterials require detailed information about the building blocks before they can be reconfigured into a desired state. Despite many realizations, a useful and simple model for these networks is as a collection of coupled mechanical mass-spring resonators (Fig. 1a). Using this model, the building blocks are defined locally by the elasticity, mass, and damping of each resonator, while the connectivity is captured by coupling springs. For a linear response, the resonator network is governed by the equation of motion

1 $$\varvec{M}\ddot{\vec{x}}+\varvec{B}\dot{\vec{x}}+\varvec{K}\vec{x}=\vec{F}$$

where ${\varvec{M}}, {\varvec{B}},$ and ${\varvec{K}}$ are the mass, damping, and elasticity matrices, respectively, and $\vec{F}$ is the external force. Here, we assume linear elasticity and aim to solve the inverse problem. Typically, the mechanical elements (${\varvec{M}}, {\varvec{B}},{\varvec{K}}$, and $\vec{F}$) of these systems are characterized by analyzing amplitude and phase spectra with non-linear least squares (NLLS), where the mechanical elements are fitting parameters. NLLS fitting requires both solving the coupled differential equations (i.e., determining a closed-form solution for $\vec{x}(\omega )$) and a priori knowledge in the form of initial guesses for each of the network’s mechanical parameters. When conducting numerical optimization, initial guesses are crucial, since the solution may converge to a local minimum determined by the initial guesses, and poor initial guesses can lead to inaccurate estimates of the true state of the network^12^. Moreover, as the number of parameters increases, the optimization problem becomes more complex, and the number of potential local minima generally increases, making NLLS more inaccurate^12^.

Recently, we developed an alternative, non-regressive algebraic approach to circumvent the issues of NLLS^12^, a method we call Network Mapping and Analysis of Parameters, or NetMAP^13^. In this approach, we transform Eq. (1) into

2 $$\varvec{\mathcal{M}}(\omega)\vec{Z}(\omega )=\vec{f}$$

where $\varvec{\mathcal{M}}(\omega)=-{\omega}^{2}{\varvec{M}}+{\mathrm{i}}\omega \varvec{B}+{\varvec{K}}$, $\vec{f}$ is the amplitude of $\vec{F}$, and $\vec{Z}(\omega )$ is the complex response vector of the network, which includes the amplitude and phase for each resonator in the network measured at a frequency $\omega$ (Fig. 1b). We then measure and construct $\vec{Z}(\omega )$ at two or more values of $\omega$, and use these measurements to rearrange Eq. (2) into an augmented homogeneous equation

3 $$\varvec{\mathcal{Z}} \vec{p}=\vec{0}$$

where $\varvec{\mathcal{Z}}$ is a matrix of known, measured quantities determined by $\vec{Z}(\omega )$. The vector $\vec{p}$ consists of the desired, unknown elements of ${\varvec{M}}\text{, }{\varvec{B}}\text{, }{\varvec{K}}$, and $\vec{f}$ (Fig. 1b, and see Supplementary Information section 2), and the solution space for $\vec{p}$ is the null-space of $\varvec{\mathcal{Z}}$. Whereas solving the equations of motion (shown as the bottom red arrow in Fig. 1b) allows us to obtain the spectrum of each resonator from the parameters, the purpose of NetMAP (top blue arrow in Fig. 1b) is to tackle the inverse problem: obtaining the underlying parameters from the available spectral data. This is crucial for characterizing networks by their fundamental building blocks. In contrast to NLLS, which might also obtain parameters from the available spectral data, NetMAP does not require a priori knowledge (i.e. neither exact nor approximate knowledge of the elements of ${\varvec{M}}\text{, }{\varvec{B}}\text{, or }{\varvec{K}}$) nor iterative computation, but instead solves for the vector $\vec{p}$ directly with as few as two response vector measurements ($\vec{Z}(\omega )$). In Carter et al.^13^ we used NetMAP to determine $\vec{p}$ for small clusters of graphene nanoelectromechanical (NEMS) resonators and we found excellent agreement with expected parameter values and broader spectral response.

While NetMAP is a promising new technique, there are many unanswered questions regarding its accuracy in predicting the mechanical parameters of the network: *How does the number *$n$* of response vectors, their signal-to-noise ratio (SNR), or the frequency at which they were measured affect the accuracy of NetMAP? How do the actual values of the mechanical parameters or the dimension of the solution space of Eq. *(3)* affect the accuracy?* To answer these fundamental questions, we simulate noisy response vectors for single-resonator (monomer) and coupled-pair (dimer) network clusters using predetermined mechanical parameters and then we use NetMAP to predict these parameters. We find that the accuracy of NetMAP predictions improves with the SNR, as does measuring response vectors near spectral peaks. While measuring additional vectors moderately improves accuracy, we find a minimum of two response vectors is sufficient, thus requiring a remarkably small number of measurements. Moreover, the dependence of the accuracy on the null-space dimension is nuanced; the accuracy of a 1D null-space solution varies with the input parameters.

## Validation approach

To assess the accuracy of NetMAP, we statistically compare the input parameters to the recovered parameters and the actual simulated spectra to the expected spectra from the model. The steps of the validation process are shown in Fig. 1c (see Supplementary Information for details.) In the first step, we choose the size of mass and spring network to model (${N}_{{\text{cluster}}}$). In this work, we modeled the monomer (a single resonator, ${N}_{{\text{cluster}}}=1$) and the dimer (two coupled resonators, ${N}_{{\text{cluster}}}=2$). We then set the network parameters ${\vec{p}}_{{\text{in}}}$ to fixed numerical values (step 2) and input them into the analytical solution of the equations of motion (EOM) to obtain an exact, noise-free complex response function for each resonator, ${z}_{i}(\omega)$ (see Supplementary Information). The response vector $\vec{z}(\omega)$ solves Eq. (2) exactly and has ${N}_{{\text{cluster}}}$ complex components.

To simulate a random experiment (step 3), we add real and imaginary noise to each response component to obtain ${Z}_{i}(\sigma , \omega )={z}_{i}(\omega)+{\Gamma }_{x,i}(\sigma , \omega)+{\text{i}}{\Gamma }_{y,i}(\sigma ,\omega )$, where $\Gamma (\sigma , \omega )$ is a pseudorandom number drawn from a zero-centered normal distribution of variance ${\sigma }^{2}$ and regenerated for each frequency $\omega$. This simple noise model qualitatively agrees with experimental data in Carter et al.^13^. Next (step 4), we select a set of noisy response vectors $\vec{Z}(\sigma, \omega)$ at $n$ discrete frequencies (*e.g.* ${\omega }_{a}$ and ${\omega }_{b}$ in Fig. 1c, so $n=2$). Each $\vec{Z}(\omega )$ provides two vector equations corresponding to the real and imaginary parts of Eq. (2), for a total of $2n{N}_{{\text{cluster}}}$ linear equations, which we use to populate the matrix elements of $\varvec{\mathcal{Z}}$. The matrix $\varvec{\mathcal{Z}}$ has dimensions $2n{N}_{{\text{cluster}}}\times N$, where $N=\dim(\vec{p})$. (See Supplementary Information sections 3.2 and 4.2 for general closed form of $\varvec{\mathcal{Z}}$ for the monomer and dimer, respectively).

To obtain the predicted parameters vector $\widehat{\vec{p}}$ (step 5), we use the simulated $\varvec{\mathcal{Z}}$ to solve $\varvec{\mathcal{Z}} \vec{p}=\vec{0}$ algebraically. An algebraic approach is not standard but is straightforward for describing resonator systems. Our solution, NetMAP, uses singular value decomposition (SVD), an algebraic approach to solving systems of equations with applications in medical imaging^14^, antenna arrays^15^, planar transmission lines^16^, and movie recommendations^17^. SVD is similar to eigensystem solvers, with singular values instead of eigenvalues and singular vectors instead of eigenvectors, while allowing the matrix $\varvec{\mathcal{Z}}$ to be rectangular rather than square. In using SVD to solve $\varvec{\mathcal{Z}}\vec{p}=\vec{0}$, we seek a singular value of zero and its corresponding singular vector, which is a solution for the physical parameters $\vec{p}$. The solution space for non-trivial $\widehat{\vec{p}}$ will be at minimum one-dimensional (1D), but may have higher dimension. The solution-space dimension provided by SVD is open to interpretation, but commonly determined by the number of singular values with $\lambda \ll 1$. We explore the accuracy of NetMAP with 1D, 2D, and 3D solution spaces. When we define higher-dimension solution spaces, we use the smallest $\lambda$ and their corresponding singular vectors ${\widehat{\vec{p}}}_{\lambda }$ in order of increasing value. In the 1D case, we scale $\widehat{\vec{p}}$ so that the force $\hat{f}$ equals the input force $f$. For higher-dimensional solution spaces, we supply additional parameter constraints (see Supplementary Information section 2.5).

Finally, to test the NetMAP parameter predictions, we replicate steps 3–5 of the simulation for a total of 1000 runs to generate a sample distribution for $\widehat{\vec{p}}$, and then use a one-sample statistical $t$-test,

$${t}_{0}\equiv \frac{\left|{\left({p}_\text{in}\right)}_{j}-{\hat{p}}_{j}\right|}{{s.e.}_{j}},$$

to quantify the agreement between the $j$th predicted network parameter ${\widehat{p}}_{j}$ and the corresponding input parameter ${p}_{j,\mathrm{ in}}$. For each $\widehat{\vec{p}}$ from the sample, we also compute the fractional error for each parameter ${e}_{j}=\left|{\widehat{p}}_{j}-{{(p}_{{\text{in}}})}_{j}\right|/{{(p}_{{\text{in}}})}_{j}$, and we calculate the correlation coefficient (${R}_{i}^{2}$) between the expected spectra $\hat{{Z}_{i}}(\omega )$ and the simulated noisy spectra ${Z}_{i}\left(\omega \right)$.

## Case studies

### A monomer

To demonstrate NetMAP’s algebraic approach, we first consider a test case with a lightly damped monomer. For a general monomer, the noisy spectrum is given by

$$Z\left(\sigma ,\omega \right)=\frac{f}{-{\omega }^{2}m+{\text{i}}\omega b+k}+\Gamma \left(\sigma ,\omega \right)+\mathrm{i\Gamma }\left(\sigma ,\omega \right).$$

**Figure 2:**
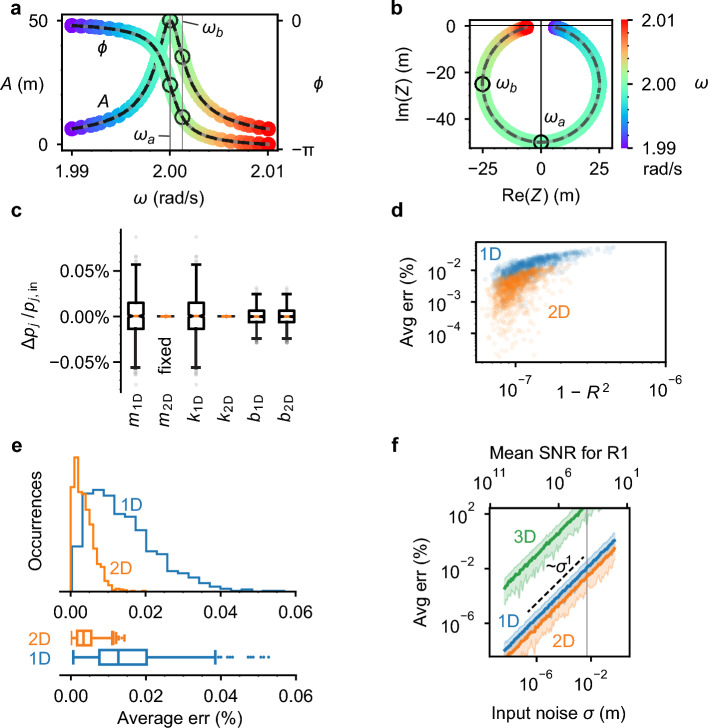
(**a**) Amplitude $A\left(\omega \right)$ and phase $\phi (\omega )$ spectrum of the lightly damped monomer, with input parameters: mass $m=4 \;\mathrm{ kg},$ damping coefficient $b=0.01\;\frac{{\text{N}}}{{\text{m}}/{\text{s}}},$ spring stiffness $k=16$ N/m, and force amplitude $f=1$ N. A subtle grey curve shows the exact spectrum while the datapoints in color show simulated spectrum measurements with standard deviation $\sigma =0.005$ m. The black dashed line shows the output spectra $\hat{A}(\omega )$ and $\hat{\phi }(\omega )$. The color scale corresponds to (**b**). (**b**) The complex amplitude $Z=A{e}^{i\phi }$ is plotted in the imaginary plane, where the datapoints are phasors, $A$ is the distance from the origin, and phase $\phi$ is the polar angle. These are the same data as (**a**). Two measurements [$Z({\omega }_{a})$ and $Z({\omega }_{b})$, where ${\omega }_{a}=2.0000 \; \mathrm{ rad}/{\text{s}}$ and ${\omega }_{b}=2.0013 \; \mathrm{ rad}/{\text{s}}$, black circles] are selected for input to NetMAP: one at the frequency of maximum amplitude and the other at a frequency corresponding to $\phi =-\frac{3}{4}\pi$. The remaining $Z(\omega )$ [colorful datapoints] are used only for validation, not for finding the recovered parameters. (**c**) Box and whisker plot showing the spread in recovered parameters, $\Delta {p}_{j}/{p}_{j,{\text{in}}}=({\widehat{p}}_{j}-{p}_{j,{\text{in}}})/{p}_{j,{\text{in}}}$, for 1D and 2D solutions with 1000 runs of the same parameters. For 2D-SVD, additional a priori information is required: we fix $m={m}_\text{in}$ in order to select the solution from the 2D solution space. (**d**) Error $\langle e\rangle$ and $1-{R}^{2}$ are correlated (1000 runs). We calculate $\langle e\rangle$ from a priori information, but we may predict it approximately from the ${R}^{2}$ value. (**e**) Histogram and box plots of the percent error $\langle e\rangle$ (1000 runs), showing a half-normal distribution. (**f**) Expanding to a range of input noise $\sigma$ for this lightly damped monomer (80 runs per $\sigma$), we find that the 2D-SVD solution is slightly more accurate than the 1D-SVD solution. The 3D solution is many orders of magnitude less accurate. The vertical grey line shows the input noise $\sigma =0.005$ m appearing in other subfigures (**a**–**e**) of this figure. The average percent error and the standard deviation are related by a power law. The average curve is calculated as a mean of the logarithm of the average errors and the shaded regions indicate 95% of the runs.

For this test case, we arbitrarily choose ${\vec{p}}_{{\text{in}}}$ with parameters: mass $m=4\text{ kg},$ damping coefficient $b=0.01$ N/(m/s), spring stiffness $k=16$ N/m, and force amplitude $f=1$ N. Moreover, we set the input error for this demonstration to $\sigma =5\times {10}^{-3}\text{ m}$. Figure 2a shows a simulated spectrum for the amplitude ($A$) and phase ($\phi$) of $Z\left(\sigma ,\omega \right)$; Fig. 2b shows the real and imaginary parts of the same $Z\left(\sigma ,\omega \right)$ plotted in the complex plane. To obtain $\widehat{\vec{p}}$, we use $Z\left(\sigma ,\omega \right)$ evaluated at the two frequencies ${\omega }_{a}$ and ${\omega }_{b}$ shown in Fig. 2a,b, which we then use to construct $\varvec{\mathcal{Z}}$. For a monomer sampled at two frequencies, the equation $\varvec{\mathcal{Z}} \vec{p}=\vec{0}$ written explicitly is:

4 $$\left(\begin{array}{lrlr}-{\omega }_{a}^{2} X({\omega }_{a})& -{\omega }_{a}Y({\omega }_{a})& X({\omega }_{a})& -1\\ -{\omega }_{a}^{2} Y({\omega }_{a})& {\omega }_{a}X({\omega }_{a})& Y({\omega }_{a})& 0\\ -{\omega }_{b}^{2} X({\omega }_{b})& -{\omega }_{b}Y({\omega }_{b})& X({\omega }_{b})& -1\\ -{\omega }_{b}^{2} Y({\omega }_{b})& {\omega }_{b}X({\omega }_{b})& Y({\omega }_{b})& 0\end{array}\right)\left(\begin{array}{c}m\\ b\\ k\\ f\end{array}\right)=\left(\begin{array}{c}0\\ 0\\ 0\\ 0\end{array}\right)$$

where $X\left(\omega \right)={\text{Re}}(Z\left(\omega \right))$ and $Y\left(\omega \right)={\text{Im}}(Z(\omega )$. The simplest way to build $\varvec{\mathcal{Z}}$ is to read the values from complex plane plots, as in Fig. 2b. By solving Eq. (4) with SVD, we obtain solutions for the physical parameters $\hat{m}$, $\hat{b}$, and $\hat{k}$. The observed $t$-statistics for 1D, 2D, and 3D solution spaces is shown in Table 1. Given our large sample size (1000), ${t}_{0}$ approximates the number of standard error intervals the predicted value deviates from the expected value. All $p$-values exceed the $\alpha =0.05$ by at least a factor of $10$, so we conclude all NetMAP predicted parameters for this trial agree with the set input values ${\vec{p}}_{{\text{in}}}$.

**Table 1:** Observed $t$-statistics and associated $p$-values for network parameters for the monomer shown in Fig. 2.

${\widehat{p}}_{j}$	${t}_{0}\equiv \frac{\left|{({p}_{{\text{in}}})}_{j}-{\hat{p}}_{j}\right|}{{s.e.}_{j}}$	$p$-value
${\hat{m}}_\text{1D}$	$0.0803$	$0.9360$
$\hat{b}_\text{1D}$	$0.2961$	$0.7672$
${\hat{k}}_\text{1D}$	$0.0805$	$0.9358$
${\hat{b}}_\text{2D}$	$0.2961$	$0.7672$
${\hat{k}}_\text{2D}$	$0.3719$	$0.7100$
${\hat{b}}_\text{3D}$	$0.3657$	$0.7146$

Results for the 1D, 2D, and 3D solution spaces are shown.

A useful measure of the accuracy of NetMAP is the fractional discrepancy $\Delta {p}_{j}/{{(p}_{{\text{in}}})}_{j}=({\widehat{p}}_{j}-{{(p}_{{\text{in}}})}_{j})/{{(p}_{{\text{in}}})}_{j}$. The $\Delta {p}_{j}/{{(p}_{{\text{in}}})}_{j}$ distributions for the 1D and 2D null-spaces are shown in Fig. 2c. The 1D result recovers all parameters within 0.04% of the input values in 95% of the trials (Fig. 2c). The 2D discrepancy results are similar or better than the 1D; for example, the 95% confidence range for the elasticity is $\sim {2.5\times 10}^{-5} \%$. The 95% confidence interval for the mean $\Delta {p}_{j}/{{(p}_{{\text{in}}})}_{j}$ are much tighter; for the 1D mass, $\frac{\Delta {m}_\text{1D}}{{m}_{{\text{in}}}}=(-4.9\pm 6.8)\times {10}^{-4} \%$.

As an additional test of NetMAP accuracy, we compare the predicted $\hat{Z}(\omega )$ to the noisy simulated $Z(\sigma ,\omega )$ with correlation analysis. $\hat{Z}(\omega )$ is shown as a black-dashed curve in Fig. 2a,b. Using ${n}_{R}=100$ simulated spectral data points, we compute the correlation coefficients ${R}_{X}^{2}$ and ${R}_{Y}^{2}$, where $X={\text{Re}}(Z)$ and $Y={\text{Im}}(Z)$. For the data in Fig. 2b, the deviation of the ${R}^{2}$ values from unity are $1-{R}_{X}^{2}=8.6\times {10}^{-8}$ and $1-{R}_{Y}^{2}=8.0\times {10}^{-8}$, or an average of $1-{R}^{2}=8.3\times {10}^{-8}$. These ${R}^{2}$ values indicate that the NetMAP prediction accounts for essentially all the variation of simulated spectra, despite calculating $\hat{Z}(\omega )$ from response vectors at just $n=2$ frequencies.

The fractional error, ${e}_{j}=\left|{\widehat{p}}_{j}-{{(p}_{{\text{in}}})}_{j}\right|/{{(p}_{{\text{in}}})}_{j}$ is a useful metric to assess the accuracy of NetMAP. However, an experimentalist without knowledge of input parameters cannot calculate the fractional error but they can calculate the coefficient of determination ${R}^{2}$. To elucidate the relationship between the average ${R}^{2}$ and ${e}_{j}$, we plot the average error $\langle e\rangle =\frac{1}{N-D}\sum {e}_{j}$ versus $1-{R}^{2}$ for each of the 1000 simulated trials and for the 1D and 2D null-spaces (Fig. 2d). For both dimensions, we observe a linear correlation (${R}_\text{1D}^{2}=0.67,\;{R}_\text{2D}^{2}=0.4$). For the 1D case, we observe $\langle e\rangle$ decreases as $\sqrt{1-{R}^{2}}$ (${R}_{1{\text{D}}}^{2}\sim 0.74$, $p\ll 0.001$). The correlation between $1-{R}^{2}$ and error provides a means for an experimentalist to assess the accuracy of recovered values without a priori information. The corresponding distributions for error $\langle e\rangle$ are provided in Fig. 2e. While the 2D error is lower than the 1D (${\langle e\rangle }_\text{1D}=0.0144\pm 0.0003\%$ vs. ${\langle e\rangle }_\text{2D}=0.0039\pm 0.0001\%$), we had to specify two input parameters ${{(p}_{{\text{in}}})}_{j}$ to solve for $\widehat{\vec{p}}$. In general, for a D-dimensional null-space, $D$ number of input parameters ${\vec{p}}_{{\text{in}}}$ are required to obtain a solution, which is a disadvantage in terms of a priori knowledge. Generally, 1D solutions are preferable and sufficient to recover the parameters with low error.

So far, we have presented results for a fixed input noise ($\sigma =5\times {10}^{-3}\text{ m}$.). To determine how the level of noise affects the error, we sweep $\sigma$ through several orders of magnitude and run the simulation 80 times per noise value. The error $\langle e\rangle$ vs. $\sigma$ is shown in Fig. 2f, with the $\sigma =5\times {10}^{-3}\text{ m}$ trial indicated with a vertical gray line. We also compute the signal-to-noise ratio defined as ${\text{SNR}}=\frac{A}{\sigma }$ and add it to the upper axis. We find that $\langle e\rangle$ for all solution space dimensions varies approximately linearly with $\sigma$ (*e.g.* for 1D, $\langle e\rangle =\beta {\sigma }^{\alpha }$ with $\alpha =1.0004\pm 0.0016$). The 2D-SVD error is the lowest, but is followed closely by the 1D error. The 3D error is several orders of magnitude larger than either 1D or 2D.

### A dimer

**Figure 3:**
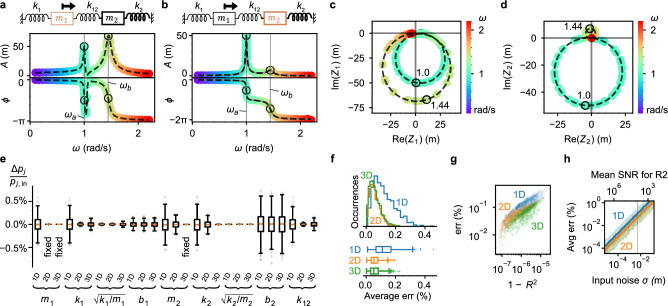
For an example dimer system with selected parameters, we demonstrate sampling at $n=2$ frequencies, each at a resonance peak (black circles in (**a**–**d**)). (**a**) Amplitude $A$ and phase $\phi$ spectra showing the motion of resonator 1 (R1). (**b**) Spectra of resonator 2 (R2). (**c**) Complex spectrum of R1, showing the same dataset as (**a**). (**d**) Complex spectrum of R2, showing the same dataset as (**b**). (**e**) Box and whisker plot showing the spread of recovered parameters as fractional discrepancy, $\Delta {p}_{j}/{p}_{j,{\text{in}}}=({\widehat{p}}_{j}-{p}_{j,{\text{in}}})/{p}_{j,{\text{in}}}$, for 1D, 2D, and 3D solutions over multiple trials. For 2D-SVD, an additional parameter, ${m}_{1}$, is fixed at ${m}_{1,in}$ in order to identify the solution within the 2D solution space. For 3D-SVD, ${m}_{2}$ is also fixed. (**f**) Histogram and box plots of the average error $\langle e\rangle$. (**g**) The average error $\langle e\rangle$ is correlated with $1-{R}^{2}$. (**h**) High SNR is key to minimizing the error. The error $\overline{\langle e\rangle }$ is proportional to the input noise $\sigma$.

We now similarly analyze a two-mass (dimer) resonator system (Fig. 3). For this system, we set the input simulation parameters as follows: ${m}_{1}= 1$ kg, ${m}_{2}= 10 {\text{ kg, }}{k}_{1}= 1$ N/m, ${k}_{2}= 10$ N/m, coupling spring ${{k}_{12}= 1\text{ N/m, }}{b_{1}= {b}_{2}= 0.1}$ N/(m/s), ${f}_{1}= 10{\text{ N}},$ and noise $\sigma =0.005 \; \mathrm{ m}.$ The force is applied to ${m}_{1}$. This case represents an anti-crossing scenario where the coupling splits the resonant frequency degeneracy. The noisy spectra of each resonator are shown in Fig. 3a–d, and we identify the two frequencies $\omega$ and corresponding responses (black circles) needed to construct $\varvec{\mathcal{Z}}$ and solve for $\widehat{\vec{p}}.$ The fractional discrepancy $\Delta {p}_{j}/{{(p}_{{\text{in}}})}_{j}$ for 1D, 2D, and 3D solutions is shown in Fig. 3e (see Supplementary Information section 4.3 for $t$-test results); we also include the discrepancy for the isolated resonance frequencies ${\omega }_{j}=\sqrt{{k}_{j}/{m}_{j}}$. We find that the damping of the undriven resonator, ${b}_{2}$, is the least accurate of the recovered parameters. Nonetheless, all parameters are recovered with an error standard deviation below 0.5%. The distributions for average fractional error $\langle e\rangle$ are provided in Fig. 3f, where ${\langle e\rangle }_{1D}=0.123\pm 0.002\%$, ${\langle e\rangle }_{2D}=0.060\pm 0.001\%$, and ${\langle e\rangle }_{3D}=0.060\pm 0.001\%$). Using $\widehat{\vec{p}}$, we calculate and plot the recovered spectra ${\hat{Z}}_{1}(\omega )$ and ${\hat{Z}}_{2}(\omega )$, shown as black dashed lines in Fig. 3a–d. As before, we plot the average error $\langle e\rangle$ versus $1-{R}^{2}$ (Fig. 3g) and the input noise $\sigma$ (Fig. 3h), and we observe a similar correlation with $1-{R}^{2}$ and functional dependence $\langle e\rangle \propto \sigma$ for all null-space dimensions (see Supplementary Information).

## Optimizing frequency selection

We have thus far demonstrated NetMAP on a monomer and a dimer system using the minimum number ($n=2$) of response vector measurements. Even in the presence of noise, the 1D null-space solution recovers all network parameters with high accuracy. While any choice of at least two frequencies for response vector measurements will allow the analysis to proceed, we expect some frequencies to yield better results than others, and that sampling response vectors at more frequencies will yield different results. To test these ideas, we varied the number and value of the sampling frequencies for both a monomer and a dimer system while characterizing the accuracy of NetMAP.

### Monomer frequency optimization

**Figure 4:**
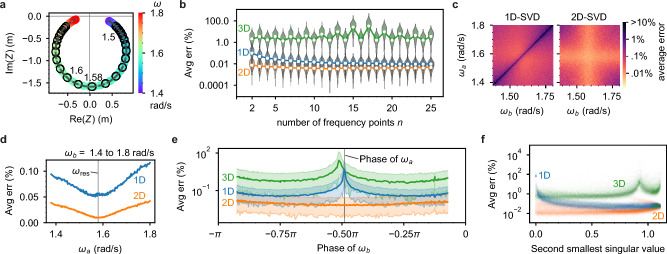
Comparing various choices of frequency measurements for analyzing a monomer with $m = 4$ kg, $b = 0.4$ $\frac{{\text{N}}}{{\text{m}}/{\text{s}}}$, $k = 10$ N/m, $f = 1$ N, resonance frequency ${\omega }_{{\text{res}}}=1.58$ rad/s, input noise $\sigma = 0.0005$ m. (**a**) Input (circled datapoints) and output (dashed black curve) spectrum for a simulated monomer with 25 circled points used for analysis. (**b**) To compare the number $n$ of frequency measurements, we plot the average error for *m,k,b* as a function of the number of frequency points used in the SVD analysis. The lines show the mean error $\overline{\langle e\rangle }$ of 100 simulated trials (shown as datapoints) for each number of frequency points. Violin plots show the distribution of $\langle e\rangle$. (**c**–**f**) Comparing the choice of frequencies if there are $n=2$ frequencies. (**c**) The average error of the SVD solution varies with the choice of the two frequencies ${\omega }_{a}$ and ${\omega }_{b}$. The 1D solution fails when the two frequencies are the same. Both the 1D and 2D solution have better results when the measurements are taken near the peak of resonance. (**d**) The average error varies with the measured frequency ${\omega }_{a}$. Here the average is taken over all the results shown in (**c**), with ${\omega }_{b}$ varying from 1.4 to 1.8 rad/s. (**e**) For a double ($n=2$ frequency) measurement of a monomer, the average error $\overline{\langle e\rangle }$ varies with the choice of frequencies measured. In this case, we fix ${\omega }_{a}$ at the resonance peak and sweep ${\omega }_{b}.$ We find that the optimum second frequency occurs when the phase at ${\omega }_{b}$ is near ${\phi }_{b}=-\frac{\pi }{4}$ or ${\phi }_{b}=-\frac{3\pi }{4}$. The colored bands correspond to 95% of the solutions. (**f**) The second smallest singular value ${\lambda }_{2}$ predicts the average error of the SVD solutions across all combinations of frequencies from trials shown in (**c**).

In order to assess the accuracy as a function of the number of measurement frequencies $n$, we first consider a moderately damped ($Q=16$) monomer with $m=$ 4 kg, $b=0.4$ N/(m/s), $k=10$ N/m, $f=1$ N, $\sigma = 5\times {10}^{-3}$ m and we sweep $n$ from $n=2$ to 25. We start with two points near the resonance frequency ${\omega }_{{\text{res}}}=1.58$ rad/s and add additional points sequentially to the right (i.e. higher frequency) and left (lower frequency) of the peak, with $\Delta \omega =0.01$ rad/s separation between measurement points (Fig. 4a, see Supplementary Information section 3.3 for the amplitude and phase spectra.). We replicate a given $\omega$ for a total of 100 runs. The SNR for the peak datapoint is $A/\sigma = 3164$. Figure 4b shows the average error $\langle e\rangle$ vs. $n$ for 1D, 2D, and 3D null-space solutions, which we label as 1D-SVD, 2D-SVD, etc. For this monomer system, the 2D-SVD solution (orange curve in Fig. 4b) has the least error, then the 1D-SVD (blue), and the 3D-SVD (green) has the largest error. The average percent error of the parameters $\vec{p}$ varies with the number of frequency points used in the SVD analysis. For this analysis, we add the frequency points in a specific order, starting with two points at each of the two amplitude peaks and sequentially adding points to the left and right of the peaks. The specific datapoints used for Fig. 4b are indicated in Fig. 4a. The 1D error gradually decreases with $n$, but with diminishing returns as the number of measurement points becomes large; the 1D mean errors for $n\ge 7$ are statistically equivalent (see Supplementary Information section 3.4 for ANOVA post hoc analysis). The 2D error dependence on $n$ is similar to the 1D, but plateaus after $n=4$. However, while the 2D solution has lower error than the 1D for low $n$, the two solutions appear to converge for $n\ge 9$; at $n=25$ the mean difference between 1 and 2D error is $(0.008\pm 0.002)\%$. As with the first monomer test case, the 3D error is orders of magnitude larger than 1D or 2D error and does not decrease with $n$ but instead oscillates with $n$ as frequency points are incorporated to the left and right. Due to the high error of the 3D solution (sample distribution, ${\langle e\rangle }_{3D}=(8\pm 15){\%}$ and see green curve in Fig. 4b), it is not recommended for this system.

To investigate the effect of frequency choice on the error, we consider two response vectors with frequencies ${\omega }_{a}$ and ${\omega }_{b}$ and measure $\overline{\langle e\rangle }$ for 1D and 2D null-spaces as we vary the frequencies across resonance. We plot $\overline{\langle e\rangle }$ vs. $({\omega }_{a}$, ${\omega }_{b})$ in Fig. 4c, and find that both the 1D and 2D solution are optimized near the peak resonance frequency (${\omega }_{{\text{res}}}=1.58$ rad/s). The “cross” patterns indicate that if either ${\omega }_{a}$ or ${\omega }_{b}$ is on resonance, then the other frequency can take nearly any value and the error remains small. For the 1D case, however, there is a narrow diagonal line of high error ($68.3\%\pm 1.2\%$ standard deviation), indicating that ${\omega }_{a}$ and ${\omega }_{b}$ must differ to obtain a low-error 1D solution. This diagonal line is absent in the 2D case, so that replicated $\vec{Z}(\omega )$ with the same frequency are possible and yield low error. To observe more general trends, we plot the average error for different choices of ${\omega }_{a}$ while taking an average of all ${\omega }_{b}$ values (Fig. 4d). This result shows that the optimal ${\omega }_{a}$ to extract $\vec{Z}({\omega }_{a})$ is near the resonant frequency, ${\omega }_{{\text{res}}}$. Moreover, by fixing ${\omega }_{a}={\omega }_{{\text{res}}}$ we determine which ${\omega }_{b}$ will yield the lowest error. Figure 4e shows the average error vs. the complex phase of the response vector $\vec{Z}({\omega }_{b})$ for varying ${\omega }_{b}.$ From this plot, the error of the 1D solution reaches a minimum near ${\phi }_{b}=-3\pi /4$ and ${\phi }_{b}=-\pi /4$, and is greatest at ${\phi }_{b}={\phi }_{a}\approx -\pi /2$. In accord with Fig. 4c, the error of the 2D solution is minimum near ${\phi }_{b}={\phi }_{a}\approx -\pi /2$. We see similar optimal phase choices for other monomer examples (see Supplementary Information section 6).

For the monomer under study, the 2D null-space solution generally has the lowest error. However, increasing the dimension of the solution space assumes the singular value of the additional degree of freedom is sufficiently small, i.e. $\lambda \ll 1$. If this value is not small, the lower dimension null-space should have lower error because the singular vector corresponding to the large singular value does not solve the homogeneous Eq. (3). To test this idea, we plot the average error for 1D, 2D, and 3D null-spaces against the value of the second smallest singular value (${\lambda }_{2}$) for each solution, as shown Fig. 4f (for this plot, we use the full set of swept frequency pairs from Fig. 4c). We find that the 2D solution, which uses both the first and second singular vectors, is more accurate when ${\lambda }_{2}$ is small, while the 1D solution improves in accuracy as ${\lambda }_{2}$ grows (see Supplementary Information section 3.4 for the 1D solution error as a function of both ${\lambda }_{1}$ and ${\lambda }_{2}$). The 3D solution additionally uses the singular vector corresponding to the third singular value (${\lambda }_{3}\ge {\lambda }_{2})$. As expected, the 3D solution has consistently higher error than either 1D or 2D.

Our monomer case studies shed light on NetMAP best practices. For the above case study, the error generally decreases modestly as the number $n$ of measured response vectors increase; before plateauing, the 1D error decreases by $\sim 5\times$ with $n=7$ and the 2D error decreases by $\sim 1.5\times$ with $n=4$. In either case, $n=2$ suffices. The 1D solution is preferred to the 2D because it requires minimal prior knowledge and has reasonably low error (i.e. $<1\%$). However, the 2D error is consistently lower (mean of $\sim 8.3\times$ across all $n$) if ${\lambda }_{1}\sim {\lambda }_{2}\ll 1$. To obtain the lowest error with the 1D solution, $\vec{Z}(\omega)$ should be measured on resonance (${\phi }_{a}\approx -\pi /2$) and $\pi /4$ radians off resonance, where ${\phi }_{b}=-3\pi /4, -\pi /4$. The sampling frequencies from Fig. 2 were selected in this way. Finally, given the error is proportional to the noise, it is beneficial to perform noise-filtered, long-integration experimental measurements of $Z\left({\omega }_{a}\right)$ and $Z\left({\omega }_{b}\right)$.

### Dimer frequency optimization

**Figure 5:**
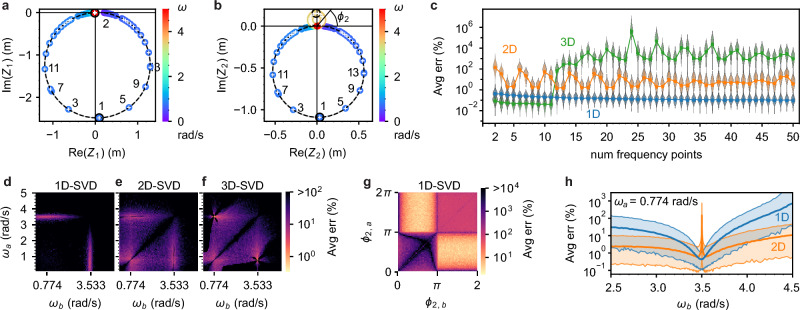
Analysis of optimal frequencies for NetMAP with an example two-mass (dimer) resonator system, with ${m}_{1,{\text{in}}}=8$ kg, ${m}_{2,{\text{in}}}=1$ kg, ${k}_{1,{\text{in}}}=2\;\frac{{\text{N}}}{{\text{m}}}$, ${k}_{12,{\text{in}}}=5\;\frac{{\text{N}}}{{\text{m}}}$, ${k}_{2,{\text{in}}}=7\;\frac{{\text{N}}}{{\text{m}}}$, ${b}_{1,{\text{in}}}=0.5\;\frac{{\text{N}}}{{\text{m}}/{\text{s}}},\;{b}_{2,{\text{in}}}=0.1\;\frac{{\text{N}}}{{\text{m}}/{\text{s}}}$, ${f}_{1}=1\text{ N}$, and $\sigma =5\times {10}^{-5}\text{ m}.$ **a,b)** Complex spectrum of R1 (**a**) and R2 (**b**), showing the simulated spectrum with noise (colorful datapoints) and the spectra recovered parameters $\hat{Z}(\omega )$ (black dashed line, for 1 trial). White plus signs indicate datapoints that are added as $n$ increases from 2 to 50, and numbers indicate the order in which the points are included, with odd numbers for the larger peak and even numbers (not all shown) for the smaller peak illustrating how we alternate adding the frequency points. Only one resonant frequency, ${\omega }_{{\text{res}}}=0.774\text{ rad/s}$ (blue points), appears in the spectrum of R1 (**a**), but a small second peak at ${\omega }_{{\text{res}}}=3.501\text{ rad/s}$ (yellow points) appears in the spectrum of R2 (**b**). (**c**) The average error varies with the number of datapoints $n$ used in the analysis, with 1D-SVD (blue), 2D-SVD (orange), and 3D-SVD (green). Simulated with 99 trials per each of 49 values of $n$, totalling 4851 trials. (**d**) If $n=2$ datapoints are used, the 1D solution is most accurate when a measurement is taken at each of the two resonant peaks. (**e**,**f**) The accuracy of the 2D and 3D solutions also varies with the pair of frequencies chosen. In general, it is less accurate for the two frequencies to be equal or nearly equal (dark diagonal lines). (**g**) Replotting (**d**) in terms of R2 phase rather than frequency shows greater detail. Subfigures (**d**–**g**) are simulated with 240,000 trials. (**h**) When ${\omega }_{a}$ is fixed at the lower resonant peak, ${\omega }_{a}=0.774$ rad/s, then the accuracy of the 1D solution is optimized when ${\omega }_{b}$ is at the higher resonant peak, 3.533 rad/s (blue dip). The 2D solution is not accurate when each measurement is at one of the two resonant peaks (sharp orange peak). Shaded areas show 95% of simulated $\langle e\rangle$ results and lines show $\overline{\langle e\rangle }$. Simulated with 1600 trials per each of 199 values of ${\omega }_{b}$, totalling 318,400 trials.

We now repeat the optimal-frequency analysis for a dimer system with the following input parameters: ${m}_{1}= 8$ kg, ${m}_{2}= 1$ kg, ${k}_{1}= 2$ N/m, ${k}_{2}=7$ N/m, coupling spring ${k}_{12}=5$ N/m, ${b}_{1}= 0.5$ N/(m/s)$, {b}_{2}= 0.1$ N/(m/s), ${f}_{1}= 1{\text{ N}},$ and noise $\sigma =5\times {10}^{-5}\text{ m}.$ Oscillating force is applied to ${m}_{1}$. The simulated spectra are shown in Fig. 5a,b (see Supplementary Information section 4.5 for corresponding amplitude and phase spectra); the spectral peaks inferred from the ${Z}_{2}\left(\omega \right)$ spectrum are at ${\omega }_{{\text{res}}}=0.774$ rad/s and ${\omega }_{{\text{res}}}=3.501$ rad/s. The higher frequency peak of ${Z}_{2}\left(\omega \right)$ has a markedly smaller maximum amplitude. The average errors for up to $n=50$ sampled response vectors are shown in Fig. 5c. The error for 1D solutions starts at ${\overline{\langle e\rangle } }_{1{\text{D}}}=(0.50\pm 0.30)\%$ (standard deviation, SD) for $n=2$, and improves with increasing $n$ down to ${\overline{\langle e\rangle } }_{1{\text{D}}}=(0.125\pm 0.077)\%$ (SD) at $n=50$. Analyzing the 1D case with ANOVA (see Supplementary Information), we see that solutions with $n\ge 16$ have equivalent error and provide a modest $\sim 2.5\times$ error improvement over the $n=2$ solution. Compared to 1D solutions, the error for 2D solutions is higher, ranging from a minimum error of $(2.37\pm 1.88)\%$ (SD) at $n=12$ up to $(215\pm 208)\%$ (SD) at $n=2$. There is a pattern that repeats for every fourth additional frequency point: when a measurement is added near the stronger $0.774$ rad/s peak, the 2D solution improves, and when a measurement is added near the $3.501$ rad/s peak, the 2D solution becomes less accurate. For this analysis, we add the frequency points in a specific order, starting with two points at each of the two amplitude peaks and sequentially adding points to the left and right of the peaks. Some of the measurements are numbered in Fig. 5a,b to indicate the order in which they are included into Fig. 5c. The 3D solution has the overall lowest error for $2\le n\le 11$, with a minimum ${\overline{\langle e\rangle } }_{3{\text{D}}}=(0.052\pm 0.037)\%$ (SD) at $n=11$, but then suffers an abrupt loss in accuracy for $n\ge 12$ where the error jumps to $(99\pm 79)\%$ (SD). To determine the optimal frequencies, we measure the average error with $n=2$ and sweep both frequencies ${\omega }_{a}$ and ${\omega }_{b}$ across a range that covers both spectral peaks (Fig. 5d–f. Each solution space dimension has a high-error diagonal band corresponding to ${\omega }_{a}={\omega }_{b}$. For the 1D solution, the lowest error occurs when one frequency is at the top of one peak and the other frequency is at the top of the other peak. The spectral peaks have the two highest SNR values that also sample both resonances. The 2D and 3D error patterns are more complex and more forgiving in terms of frequency choice; in both cases, it is sufficient to have one frequency near a spectral peak resonance, but the lowest error still occurs by sampling near each spectral peak.

To see the detail of the 1D case, we plot the Fig. 5d dataset as a function of complex phases of ${Z}_{2}\left({\omega }_{a}\right)$ and ${Z}_{2}({\omega }_{b})$ in Fig. 5g (see Supplementary Information section 4.5 for phase spectra plots). The ${Z}_{2}(\omega )$ phase ${\phi }_{2}(\omega )$ is one-to-one, unlike ${\phi}_{1}(\omega)$, so there is no ambiguity about which value of ${\phi }_{2}$ corresponds to which value of $\omega$. The phase plot shows consistently low error for quadrants II and IV, corresponding to recommended phase values ${\phi }_{2}\in [0,\pi ]$ for one measurement and ${\phi }_{2}\in [\pi , 2\pi ]$ for the other. In terms of the complex spectrum of ${Z}_{2}(\omega )$ (Fig. 5b), these phase values correspond to the upper and lower loops (spectral peaks). High error occurs in quadrants I and III, where the two response vectors are sampled on the same loop while the other is not sampled. Moreover, dark bands of high error occur when either phase is equal to $0$ or $\pi$, which correspond to response vectors near the origin of the complex plane with near zero SNR. We further examine the behavior of the 1D and 2D solutions near the spectral peaks by fixing ${\omega }_{a}=0.774$ rad/s and sweeping ${\omega }_{b}$, as shown in Fig. 5h. For the 1D case, error decreases as ${\omega }_{b}\to 3.533$ rad/s, which corresponds to the higher frequency spectral peak, where it reaches a minimum of $0.5\%$. The 2D case has similar behavior, but spikes to a maximum over $100\%$ when ${\omega }_{b}=3.533$ rad/s, indicating a failure in the 2D null-space accuracy when the response vectors are sampled directly on resonance. Thus, for a dimer with two resonant peaks and a 2D solution, measuring near—but not on—the spectral peaks is ideal.

## Accuracy varies with input parameters

So far, we have presented case studies of monomer and dimer resonator systems with fixed input parameters, ${\vec{p}}_{{\text{in}}}$. However, it is possible that the error behavior we observe depends on the choice of ${\vec{p}}_{{\text{in}}}$. To probe the dependence of the error on the input parameters themselves, we run a full, replicated ${2}^{k}$ factorial experiment for a general dimer system by varying the mechanical parameters (see Supplementary Information section 2.6 for Factorial Methods). We screened and ranked the mechanical parameters as predictors of average error for a dimer solved using 1D-SVD; including up to two-factor interactions, the order from most predictive to least is: $f$, ${m}_{1}$, ${m}_{2},\;{k}_{12}$, and the ${k}_{1}\cdot {k}_{2}$ interaction (see Supplementary Information section 7 for full effects model results). We previously discussed using $1-{R}^{2}$ and ${\lambda }_{2}$ as indicators of average error (Figs. 3g and 4d).

**Figure 6:**
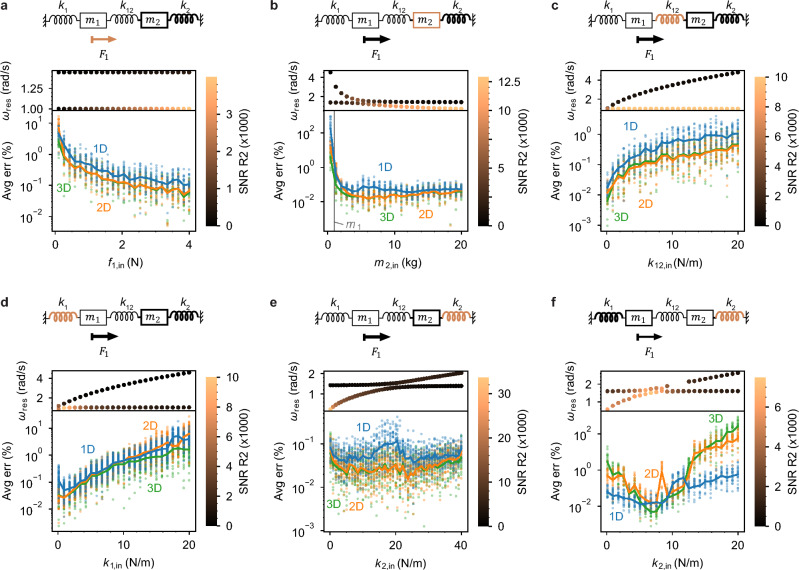
Varying dimer parameters affects the resonance frequencies and average error. In (**a**–**e**), the input parameters are ${m}_{1}=1\text{ kg}$, ${m}_{2}=10\text{ kg}$, ${k}_{1}=1$ N/m, ${k}_{12}=1$ N/m, ${k}_{2}=10$ N/m, damping ${b}_{1}={b}_{2}=0.1\;\frac{{\text{N}}}{{\text{m}}/{\text{s}}},$ and force amplitude ${f}_{1}=10$ N, except the single parameter that is varied: force amplitude in (**a**), mass 2 in (**b**), coupling spring in (**c**), spring ${k}_{1}$ in (**d**), and spring ${k}_{2}$ in (**e**). In (**f**), ${k}_{2}$ is varied and the input parameters are ${m}_{1}=5\text{ kg}$, ${m}_{2}=3\text{ kg}$, ${k}_{1}=12$ N/m, ${k}_{12}=1$ N/m, ${b}_{1}=1\;\frac{{\text{N}}}{{\text{m}}/{\text{s}}},\;{b}_{2}=0.5\;\frac{{\text{N}}}{{\text{m}}/{\text{s}}}$, and ${f}_{1}=10$ N. The resonator cartoons above each plot indicate the strength of parameters with line thickness, and the varied parameter is indicated in orange in the cartoon. The average error of the recovered parameters is plotted as a function of the varied parameter for three solution space dimensions, with 1D-SVD (blue), 2D-SVD (orange), and 3D-SVD (green). Error for individual simulations $\langle e\rangle$ is shown as datapoints and the average error across the simulations $\overline{\langle e\rangle }$ is shown as lines.

To gain a deeper understanding of how the parameters $\vec{p}$ of the resonant system affect the SVD accuracy, we plot the average error as a function of varied parameters in a few example dimer cases (Fig. 6). Each cartoon in Fig. 6 shows one parameter in orange that we vary while keeping all others fixed. We identify the two peak frequencies ${\omega }_{{\text{res}}}$ using a peak-finding function^18^ and plot them below each cartoon to illustrate their variation with the parameter. To improve the accuracy of the NetMAP results, we choose 4 additional frequencies on each side of the two peak frequencies ${\omega }_{{\text{res}}}$ for a total of $n=10$ frequencies for analysis. To demonstrate the impact of signal to noise ratio (SNR) on the error as we vary each parameter, we plot the ${\omega }_{{\text{res}}}$ datapoints with higher SNR in orange, highlighting how the error falls as SNR increases. Increasing the driving force (Fig. 6a) or the non-driven mass ${m}_{2}$ (Fig. 6b) causes the SNR to increase and the error in the recovered parameters to decrease correspondingly. This matches our findings that error is inversely proportional to SNR, and we generally find that more accurate recovered parameters result for dimer systems with high force amplitude and larger ${m}_{2}$. We observe the error increases as we increase the coupling spring stiffness ${k}_{12}$ from 0 to 20 N/m (Fig. 6c), and this is usually true for dimers. For this dimer system, a lower ${k}_{1}$ corresponds to more accuracy (Fig. 6d), but this is not generalizable to all dimer systems. As ${k}_{2}$ is varied in Fig. 6e, the resonance peaks show an anticrossing near ${k}_{2}=20$ N/m, and the 1D solution loses accuracy near the anticrossing. This is a specific result for this particular dimer system. To see how an anticrossing affects a dimer system with different input parameters, we consider the system shown in Fig. 6f, with an anticrossing near ${k}_{2}=7$ N/m, and find, in contrast, that the 1D solution has higher accuracy near the anticrossing, suggesting that anticrossings may increase or decrease the error and the variation in error is likely associated with the SNR. Furthermore, to illustrate the effect on accuracy when the peak-finder fails to identify one of the resonance peaks, we allow the peak-finder to miss the higher ${\omega }_{{\text{res}}}$ peak for ${k}_{2}=$ 10 to 12 N/m, and find that the 1D solution loses accuracy while the 2D solution gains accuracy in that range. Thus, Fig. 6 shows a detailed view of how the parameter values affect the accuracy of the algebraic solution.

The frequency optimization and factorial studies provide broader NetMAP best practices for the dimer system. Driving with a higher amplitude force improves accuracy. For our case study in Fig. 5, the error decreases modestly as the number of frequency points increases, though $n=2$ measured response vectors are sufficient. As it was for the monomer case, the 1D solution is preferred to the 2D or 3D because it requires minimal prior knowledge of the parameters, while still having reasonably low error, and because the 2D and 3D solutions have erratic accuracy, depending heavily on the particular frequency points sampled in our example in Fig. 5. To obtain the lowest error with the 1D solution, $\vec{Z}(\omega )$ should be measured at each of the two resonance peaks of the dimer spectrum. The two sampling frequencies in Fig. 3 were selected in this way. As with the monomer case, the error is proportional to the noise, so it is beneficial to perform noise-filtered, long-integration experimental measurements of $Z({\omega }_{a})$ and $Z({\omega }_{b})$.

## Discussion

For an experimentalist, NetMAP offers a means to analyze amplitude and phase data in order to reveal the physical parameters for each individual resonator and the coupling between the resonators. This phase-sensitive data is standard for lock-in amplifier measurements. However, for NetMAP to be useful, the experimentalist must measure the amplitude and phase for each resonator in the network, which in some cases may be challenging. The SNR range we describe here (e.g. ${10}^{3},$ or 30 dB SNR) is attainable to experimentalists working with a wide variety of systems, including electronic or micromechanical resonator networks. See Carter et al.^13^ for an experimental example of using NetMAP in a NEMS system.

In cases where NetMAP returns an inadequate ${R}^{2}$ value, it may be advantageous to combine NetMAP and NLLS fitting by using the results from NetMAP as the initial guesses for an iterative solution, and thereby improving ${R}^{2}$. Since ${R}^{2}$ correlates with the error in the parameters, this is expected to improve the accuracy of the results.

We have shown how well NetMAP performs for a linearized monomer (single mass) and dimer (double mass) system, but further investigation is needed to evaluate its performance in larger and nonlinear systems. Our calculations (see Supplementary Information section 2) show that, even as the number of resonators increases, only two discrete frequencies are needed for the required response vectors $\vec{Z}$. For a larger system, the size of the response vectors would scale with the number of resonators, increasing the size of the matrix $\varvec{\mathcal{Z}}$, which may require solution spaces of higher dimension. We are building an understanding of the parameter space in which NetMAP will be useful. Future work will explore NetMAP performance in larger systems using similar techniques to discover best practices for using NetMAP as the systems scale up. We anticipate that similar results may hold for larger networks, enabling characterization of larger systems. Moreover, future work will explore the use of NetMAP to extract the mechanical parameters of nonlinear resonator networks^19,20^; in one potential path, the network could first be driven in the linear regime and characterized by NetMAP. Then, the network would be driven into the nonlinear regime and characterized via traditional methods (e.g. NLLS) with a much reduced and simplified parameter space.

The potential applications of NetMAP are wide, encompassing both natural phenomena like atomic solids, the brain, and celestial bodies, as well as diverse synthetic systems, including solid-state and optical qubit arrays^10,11,21^, photonic/phononic crystals^22,23^, and neural networks^24^. These systems play a pivotal role in applications such as neuromorphic and quantum computing^10,25^, strongly correlated phases^21^, and metamaterials^26^. Consequently, there is a vibrant effort to comprehend, control, and engineer their collective behavior, and NetMAP could be beneficial. A particularly important application of NetMAP could be to a programmable NEMS network^22,23^, which is a promising testbed for generalized resonator assemblies. The aim of these programmable networks is to finely tune the resonator building blocks and coupling to modify the collective properties of the network, and thereby enable applications like reconfigurable phononic crystals^24,25^, tunable thermal transport^27,28^, computing and simulation^29,30^, and more. Recent progress in NEMS networks includes demonstrating collective phenomena in modular assemblies^7^ and lattices^27,29^ and developing tools to tune individual resonators^31^ and coupling^32–34^. However, there is a critical need for scalable, spatially resolved characterization methods to assess mechanical properties, resonator configurations, and network states, a need for which NetMAP is ideally suited.

## Conclusions

In this study, we have presented and validated NetMAP, an efficient and reliable algebraic approach for calculating the physical parameters of resonator networks. We have tested NetMAP’s accuracy for monomer and dimer systems as a function of the number of samples, choice of frequency points, and null-space dimension, and we provided a method for estimating the percent error using quantities available to an experimentalist. The spectra must be measured for each resonator at a minimum of two frequencies, ideally measured near resonance. We have developed NetMAP to measure the physical parameters of a MEMS system of coupled graphene resonators^13^. Future work includes exploring larger systems, including a two-dimensional grid of resonators; relating to other physical resonator systems, including RLC circuits; simulating nonlinear springs; and considering systems where the topology of the system is unknown. NetMAP enables the characterization of the building blocks and connectivity of a diverse array of resonator networks, which promises to enhance the ability to design, tune, and program engineered resonator networks, such as micromechanical systems, and to better understand natural resonator systems, such as neural networks.

### Supplementary Information

Supplementary Information.

## Data Availability

The simulated data supporting the findings of this study are available from the corresponding author upon reasonable request.
