# Supplementary material for: Validating an algebraic approach to characterizing resonator networks
Source: Sci Rep. 2024 Jan 15;14:1325. doi: 10.1038/s41598-023-50089-1 (PMC10789822; doi:10.1038/s41598-023-50089-1)
Supplement: Supplementary file 1 — Supplementary Information. [file 41598_2023_50089_MOESM1_ESM.pdf]

Supplementary Information: Validating an algebraic approach to characterizing resonator networks

Viva R. Horowitz [1] *, Brittany Carter [2,3,4], Uriel F. Hernandez [2,3,4], Trevor Scheuing [1], and Benjamín J. Alemán [2,3,4,5] *

[1]Physics Department, Hamilton College, Clinton, New York, 13323, United States

[2]Department of Physics, University of Oregon, Eugene, Oregon, 97403, United States

[3]Materials Science Institute, University of Oregon, Eugene, Oregon, 97403, United States

[4]Center for Optical, Molecular, and Quantum Science, University of Oregon, Eugene, Oregon, 97403, United States

[5]Phil and Penny Knight Campus for Accelerating Scientific Impact, University of Oregon, Eugene, Oregon, 97403, United States

* Corresponding authors: Viva R. Horowitz, vhorowit@hamilton.edu, Benjamín J. Alemán, baleman@uoregon.edu.

Contents

1. Symbol list ... 2
    1.1 Matrices ... 2
    1.2 Vectors of complex spectra ... 2
    1.3 Counts, indices, and more ... 3
    1.4 Force ... 4
    1.5 Results of singular value decomposition ... 4
    1.6 Error ... 4
2. Extended description of NetMAP ... 5
    2.1 Resonator network theory ... 5
    2.2 Solving the equations of motion ... 5
    2.3 Determining the number of frequencies required for NetMAP ... 6
    2.4 Reorganizing the system of linear equations ... 8
    2.5 Scaling the singular vector: the question of solution space dimension ... 9
    2.6 Factorial methods ... 11
    2.7 Simplifying assumptions ... 12
3. Monomers ... 13
    3.1 Simulating a monomer ... 13
    3.2 Recovering monomer parameters using SVD ... 14
    3.3 Details for the monomer case shown in Figure 2 of the main text ... 15

3.4 Details for the monomer case shown in Figure 4 of the main text 16
4. Dimers 19
4.1 Simulating the dimer....19
4.2 Recovering the dimer parameters with SVD....20
4.3 Statistical comparison tests for dimer case....21
4.4 Error is inversely proportional to the SNR....22
4.5 Details for the dimer case shown in Figure 5 of the main text....22
5. Experimental considerations for measuring absolute phase....25
6. Additional Cases....25
6.1 Heavily damped monomer 25
6.2 Medium-coupled dimer 27
6.3 Strongly coupled dimer....29
6.4 Force applied to both resonators of a dimer....30
7. Factorial experiments varying every parameter between two levels 31

1. Symbol list

1.1 Matrices

$\boldsymbol{\mathcal{M}}$ matrix of desired unknown values

$\boldsymbol{\mathcal{Z}}$ matrix constructed from measured or noisy simulated values of the spectrum at discrete frequency points

$\boldsymbol{M}$ mass matrix

$\boldsymbol{B}$ damping matrix

$\boldsymbol{K}$ stiffness or elasticity matrix

D = nullity of $\boldsymbol{\mathcal{Z}}$**,** or solution space dimension, where the solution space is the null-space of $\boldsymbol{\mathcal{Z}}$**.** The value of D is open to interpretation because $\mathcal{Z}$ has noise.

1.2 Vectors of complex spectra

$\vec{Z}(\omega)$: measurement vector or response vector (with noise), with elements $Z_1(\omega), Z_2(\omega), \dots Z_{N_{\text{cluster}}}(\omega)$ for each resonator i in the cluster.

$\vec{z}(\omega)$: simulated response vector (without noise) with elements $z_1(\omega), z_2(\omega), \ldots z_{N_{\text{cluster}}}(\omega)$ for each resonator in the cluster.

$\Gamma_{x,i}(\sigma, \omega)$: A pseudorandom number generated from a Gaussian distribution (standard deviation σ) to add noise to the real part of Z_i for the ith resonator at a given discrete frequency ω.

$\Gamma_{y,i}(\sigma, \omega)$: A pseudorandom number generated from a Gaussian distribution (standard deviation σ) to add noise to the imaginary part of Z_i for the ith resonator at a given discrete frequency ω.

ω angular frequency. The resonator network response frequency is assumed to be equal to the driving frequency for the steady state solution.

$\omega/2\pi$ frequency in MHz

$\vec{X}(\omega)$: The real part of $\vec{Z}(\omega)$, that is $\vec{Z}(\omega) = \vec{X}(\omega) + \mathrm{i}\vec{Y}(\omega)$

$\vec{Y}(\omega)$: The imaginary part of $\vec{Z}(\omega)$

$\vec{A}(\omega)$: The amplitude of $\vec{Z}(\omega)$, that is $Z_i(\omega) = A_i e^{\mathrm{i}\phi_i}$,

$\vec{\phi}(\omega)$: The phase of $\vec{Z}(\omega)$

1.3 Counts, indices, and more

N_{unknowns}: total number of unknown parameters

N_{cluster}: Number of oscillating masses. $N_{\text{cluster}} = 1$ for monomer, 2 for dimer

n: number of frequencies selected for analysis. If $n = 2$, we call it a double measurement.

ω_a, ω_b or $\omega_1, \omega_2, \ldots, \omega_n$: input frequencies, the set of frequencies selected for analysis.

n_t: number of trials (number of repeats + 1)

i= each resonator, from 1 to N_{cluster} (e.g. Z_i, ϕ_i, $R^2_{A,i}$, $R^2_{\phi,i}$)

i = imaginary constant

$\vec{p}$: parameters vector. It includes all unknown parameters and possibly 1 or more known parameters for scaling the solution. The order of the parameters in the parameters vector must be consistent with the order of the columns of $\boldsymbol{\mathcal{Z}}$.

$\vec{p}_{\text{in}}$: input parameters. This is the *a priori* information.

$\hat{\vec{p}}$: the parameters output by NetMAP (Network Mapping and Analysis of Parameters). Components of this vector are $\hat{m}_1, \hat{b}_1, \hat{k}_1$, et cetera.

$N = \dim(\vec{p})$ the number of parameters

j: index of each parameter from 1 to N, e.g. parameter p_j, error e_j. In Fig. 1a, $p_1 = m, p_2 = b, p_3 = k$.

$\hat{\vec{Z}}$, $\hat{\vec{A}}$, $\hat{\vec{\phi}}$, $\hat{\vec{X}}$, $\hat{\vec{Y}}$: The complex spectrum vector and related real-valued parts, calculated from $\hat{\vec{p}}$.

r: number of trials for a factorial experiment

R1: resonator 1. The resonator we are driving with an oscillating force.

R2: resonator 2 in a two-mass (dimer) system. The resonator we are not directly driving.

n_R: number of frequencies for calculating R-value

$R^2_{A_i}$: coefficient of determination comparing the amplitude spectrum $\hat{A}_i(\omega)$ to the measured amplitude $A_i(\omega)$.
R^2_{ϕ}: coefficient of determination comparing $\hat{\phi}_i(\omega)$ to $\phi_i(\omega)$
$R^2_{X_i}$: coefficient of determination comparing $\hat{X}_i(\omega)$ to $X_i(\omega)$
$R^2_{Y_i}$: coefficient of determination comparing $\hat{Y}_i(\omega)$ to $Y_i(\omega)$
R^2: The average of $R^2_{X_i}$ and $R^2_{Y_i}$, taking the average over all resonators i

k: monomer spring constant

k: number of parameters in factorial. Used in the context: 2^k.

1.4 Force

$\vec{F}$: force vector, with components for each oscillating mass. The capital notation indicates that it is time-dependent (oscillating). $\dim(\vec{F}) = N_{\text{cluster}}$

F_1 or F: the oscillating force pushing mass 1.

$\vec{f}$, force vector, just the amplitude (with the time-dependent part divided out). $\vec{F} = \vec{f}\cos\omega t$

f_1 or f, the force amplitude pushing mass 1. $F_1 = f_1 \cos\omega t$

1.5 Results of singular value decomposition

$\lambda_1, \lambda_2, \dots, \lambda_N$ the singular values of $\mathcal{Z}$, sorted from smallest to largest
$\vec{p}_1, \vec{p}_2, \dots \vec{p}_N$, the associated singular vectors
$\lambda_1, \lambda_2, \dots, \lambda_D$ the subset of singular values that correspond to the solution space
$\vec{p}_{1,\text{un}}$ the unscaled singular vector associated with the smallest singular value λ_1. It is normalized to length 1.
$\vec{p}_{2,\text{un}}$ the unscaled singular vector associated with the second smallest singular value λ_2. It is normalized to length 1.
$\hat{\vec{p}}_{1\text{D}}, \hat{\vec{p}}_{2\text{D}}, \hat{\vec{p}}_{3\text{D}}$: the recovered parameters, calculated using $D = 1$, $D = 2$, and $D = 3$, respectively.

1.6 Error

The error is a figure of merit to show how accurate recovered parameters are.

$$e_j = \frac{|\hat{p}_j - p_{j_{\text{in}}}|}{p_{j_{\text{in}}}} \cdot 100\% \qquad \text{For example, } e_m = \frac{|\hat{m} - m_{\text{in}}|}{m_{\text{in}}} \cdot 100\% \text{ is the percent error in mass.}$$

Δp: the discrepancy, $\Delta p = \hat{p}_j - p_{j,\text{in}}$. For example, $\Delta b = \hat{b} - b_{\text{in}}$.

$\bar{e}_j$: the logarithmic average of the error, taken over many trials, where j indexes the parameter (for box and whisker plots)

$\langle e\rangle = \frac{e_1+e_2+\cdots+e_N}{N-D}$ average across parameters for an individual simulation trial

$\langle\bar{e}\rangle$: logarithmic average across trials first, then arithmetic average across parameters

$\overline{\langle e\rangle}$: arithmetic average across parameters first, then logarithmic average across trials.

2. Extended description of NetMAP

2.1 Resonator network theory

A system of equations for a resonator network can be organized into the matrix form:

$$\boldsymbol{M}\ddot{\vec{x}} + \boldsymbol{B}\dot{\vec{x}} + \boldsymbol{K}\vec{x} = \vec{F}, \tag{S1}$$

where $\vec{x}$ is the displacements for each resonator, $\vec{F}$ is the oscillating forces driving each resonator, and $\boldsymbol{M}$, $\boldsymbol{B}$, and $\boldsymbol{K}$ are matrices with all information about the inertia, damping, and elasticity of the network. If the force vectors are all oscillating with driving frequency ω, then we guess an oscillating steady-state solution $\vec{x} = \vec{Z}e^{\mathrm{i}\omega t}$ where $\vec{Z}$ is a constant complex amplitude vector with components $Z_i = A_i e^{\mathrm{i}\phi_i}$, and obtain

$$-\omega^2 M\vec{Z} + \mathrm{i}\omega B\vec{Z} + K\vec{Z} = \vec{f}, \tag{S2}$$

where $\vec{f}$ is the amplitude of the force vector, $\vec{F} = \vec{f}e^{\mathrm{i}\omega t}$. More compactly, the equation of motion system can be written

$$\mathcal{M}(\omega)\vec{Z}(\omega) = \vec{f} \tag{S3}$$

where the symmetric matrix $\boldsymbol{\mathcal{M}}(\omega) = -\omega^2\boldsymbol{M} + \mathrm{i}\omega\mathbf{B} + \boldsymbol{K}$ contains complete information about the inertia, elasticity, and damping of the network.

2.2 Solving the equations of motion

In order to simulate spectra to validate NetMAP, our approach is to set the input values $\vec{p}_{\mathrm{in}}$, then calculate the spectra $Z(\omega)$ with added random Gaussian noise, thereby simulating an experiment (steps 2 and 3 of **Fig. 1c**). To calculate the input spectra, we implement Cramer's rule in SymPy[1] to symbolically solve Eq. (S2) given the input values $\vec{p}_{\mathrm{in}}$. This yields the expected response vector

$\vec{z}(\omega)$, where each vector component z_i corresponds to the ith resonator. To simulate noisy measurements, we add noise from a random Gaussian distribution to each vector component:

$$Z_i(\omega) = z_i(\omega) + \Gamma_{x,i}(\sigma,\omega) + \mathrm{i}\Gamma_{y,i}(\sigma,\omega). \tag{S4}$$

We add noise to the real and imaginary parts of z_i rather than to the amplitude and phase because doing so better matches experimental results, especially at low amplitudes, where the phase becomes highly uncertain. We also use the same solution to the equations of motion to calculate the output spectra $\hat{Z}_i(\omega)$ for step 7 of **Fig. 1c**, but we use the output parameters $\hat{\vec{p}}$ and do not add simulated noise.

While the method we present here would be appropriate for any resonator system, we consider as a particular case a micromechanical system where we measure the amplitude and phase of each resonant mass using scanning interference measurements (SIM).[2,3]

2.3 Determining the number of frequencies required for NetMAP

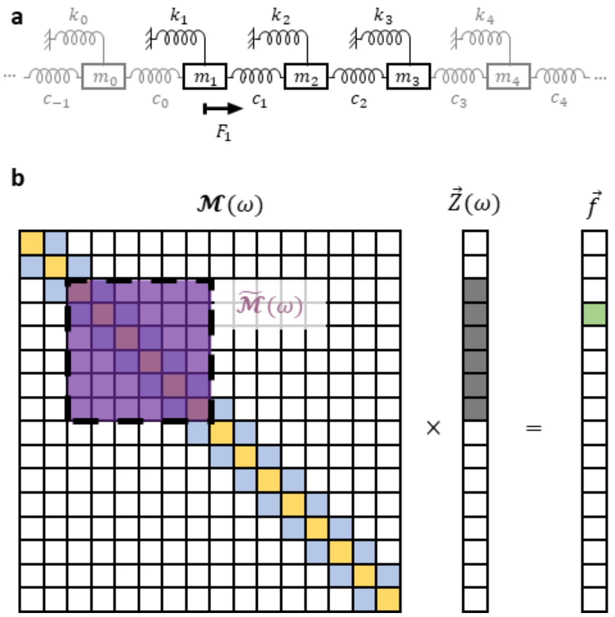

Figure S1 a) An oscillator network made up of a linear chain of masses and springs. b) Pictorial visualization of equation of motion showing the cluster response vector $\vec{Z}(\omega)$ and submatrix $\widetilde{\mathcal{M}}(\omega)$.

1. Clusters

Consider the case of a linear chain of resonators (**Figure S1a**) where we apply an oscillating force of amplitude f to one resonator in the chain. For a linear chain of resonators (**Figure S1a**), with coupling springs of stiffness c_i only between nearest neighbors, the matrix $\boldsymbol{\mathcal{M}}$ is tridiagonal (**Figure S1b, left**), with diagonal elements (yellow squares) of the form $-m_i\omega^2 + \mathrm{i}b_i\omega + c_{i-1} + c_i + k_i$ and off-diagonal elements (blue squares) of the form $-c_i$, corresponding to Eq. (S2).

In an experimental system that is driven at one resonator (**Figure S1b**, right, light green square), a finite number of resonators will respond, indicated by the shaded squares for $\vec{Z}(\omega)$, which selects a relevant submatrix $\widetilde{\boldsymbol{\mathcal{M}}}(\omega)$ (**Figure S1b**, purple square). Our response vector approach determines elements of $\widetilde{\boldsymbol{\mathcal{M}}}(\omega)$ from the observations of $\vec{Z}(\omega)$ using Eq. (S3). However, the number of unknown elements in $\widetilde{\boldsymbol{\mathcal{M}}}(\omega)$ and f is larger than the number of equations provided by a measurement of $\vec{Z}(\omega)$ at one frequency, thereby requiring multiple measurements of $\vec{Z}(\omega)$ at distinct values of ω. The number of required frequencies is determined by the number of unknowns in the network or network cluster, N_{unknowns}. Assuming a unique mass and damping for each resonator and setting the edge spring constants to zero, we write N_{unknowns} in terms of the number of resonators in the network cluster:

$$N_{\text{unknowns}} = 4 \times N_{\text{cluster}}.$$

The full set of unknown parameters include m_i, b_i, k_i, all relevant c_i's, and the amplitude f. The cluster size (N_{cluster}) is determined by the number of resonators with detectable oscillation, and will always be equal to or less than the total number of resonators in the network. Moreover, the response vector $\vec{Z}(\omega)$ will have N_{cluster} non-zero components (although some might be zero or undetectable when sampled at some frequencies) and $\widetilde{\boldsymbol{\mathcal{M}}}(\omega)$ will be a symmetric, tridiagonal $N_{\text{cluster}} \times N_{\text{cluster}}$ matrix. Each measurement of $\vec{Z}(\omega)$ at a fixed value of ω solves

$$\widetilde{\boldsymbol{\mathcal{M}}}(\omega)\vec{Z}(\omega) = \vec{f} \tag{S5}$$

and thus provides two equations per resonator (due to real and imaginary parts):

$$N_{\text{equations}} = 2 \times N_{\text{cluster}}. \tag{S6}$$

Therefore, to determine the unknowns, we only need to acquire response vectors at a minimum of two frequencies—$\vec{Z}(\omega_a)$ and $\vec{Z}(\omega_b)$. These response vectors then provide a system of $4 \times N_{\text{cluster}}$ linear equations.

In cases where the size of the resonator network is finite, we may have the cluster encompassing the entire network, such that $N_{\text{cluster}} = N_{\text{unknowns}}$. In some experimental cases, motion of some resonators at a given ω will be small and undetectable by the apparatus and require acquisition of additional response vectors $\vec{Z}(\omega)$. When a resonator's motion is undetectable at some or all driving frequencies, then the number of linear equations is reduced; if the undetectable resonator is in the interior of the cluster, we lose six equations, while if at the edge of the cluster we lose four equations. Therefore, in these cases it is important to ensure that all resonators have a measurable amplitude at the two driving frequencies, or else to measure $\vec{Z}$ at additional driving frequencies.

2.4 Reorganizing the system of linear equations.

The response vectors at $n = 2$ frequencies, $\vec{Z}(\omega_a)$ and $\vec{Z}(\omega_b)$, together yield a system of $4 \times N_{\text{unknowns}}$ linear equations composed of

$$\widetilde{\boldsymbol{\mathcal{M}}}(\omega_a)\vec{Z}(\omega_a) = \vec{f} \text{ and } \widetilde{\boldsymbol{\mathcal{M}}}(\omega_b)\vec{Z}(\omega_b) = \vec{f} \tag{S7}$$

where each component $Z_i(\omega)$ has real amplitude $A_i(\omega)$ and phase $\phi_i(\omega)$:

$$Z_i(\omega) = A_i(\omega)e^{\mathrm{i}\omega\phi_i}. \tag{S8}$$

We combine and reorganize Equations (S7) into the following single linear homogenous equation

$$\boldsymbol{\mathcal{Z}}\vec{p} = \vec{0} \tag{S9}$$

where $\boldsymbol{\mathcal{Z}}$ is a real-valued matrix with matrix elements that depend on the known measured quantities $A_i(\omega)$, $\phi_i(\omega)$, and ω (step 4 of **Fig. 1c**). See below for the form of $\boldsymbol{\mathcal{Z}}$ for the monomer and dimer case. When constructing $\mathcal{Z}$ in practice and calculating the error of $\vec{Z}(\omega)$, the vector $\vec{p}$—which we call the *parameters vector*—is an N_{unknowns} -dimensional vector composed of the unknown mechanical parameters of the cluster: m_i, b_i, k_i, all relevant c_i's, and the force amplitude vector $\vec{f}$. Including $\vec{Z}(\omega)$ at additional driving frequencies will expand the dimensionality of $\boldsymbol{\mathcal{Z}}$ and $\vec{p}$, which in general will over-determine the system of equations. Finally, we use singular value decomposition (SVD) to find the solution space of $\boldsymbol{\mathcal{Z}}$, which determines the output parameters vector $\hat{\vec{p}}$.

Here, we simulate cases where the force amplitude vector is $\vec{f} = \langle f_1, 0\rangle$, only driving the first resonator, but other force amplitudes $\langle f_1, f_2\rangle$ are possible. Sampling response vectors from at least $n = 2$ drive frequencies, we then use equations of the form Eq. (*S7*) to construct $\boldsymbol{\mathcal{Z}}$ and the system of linear equations $\mathcal{Z}\vec{p} = \vec{0}$. We apply a NumPy SVD solver[4,5] in Python to factorize and identify the solution

space of $\mathcal{Z}$, and thereby generate a solution to recover the parameters $\hat{\vec{p}}$, where the hat symbol indicates that these are values recovered by the SVD analysis, not simulated measurements (step 5 of **Fig. 1c**).

We obtain from the NumPy SVD solver the singular values $\lambda_1, \lambda_2, \dots, \lambda_N$, sorted from smallest to largest, each associated with a singular vector $\vec{p}_1, \vec{p}_2, \dots \vec{p}_N$, where $N = \dim(\vec{p}_i)$ is the number of parameters. With noise, the null value of $\boldsymbol{\mathcal{Z}}\vec{p} = \vec{0}$ is not precisely zero, so we assume the singular vector associated with the smallest singular value λ_1 corresponds to the solution space. We may also consider multiple small singular values $\lambda_1, \lambda_2, \dots, \lambda_D$ to be degenerate, even if they are not precisely equal to zero, and, in that case, we must find the solution within a D-dimensional solution space, for some positive integer $D \leq N$.

2.5 Scaling the singular vector: the question of solution space dimension

Since NumPy SVD solver provides normalized singular vectors, each of length 1, we must therefore scale the SVD output to identify the solution within the D-dimensional solution space and recover the numeric parameters. If we assume the solution space is 1D, we use $D = 1$ known values to scale the output vector. Here, we will require the force amplitude f to equal the input value f_{in}, and thus we obtain a unique scaled solution $\hat{\vec{p}}_{1\text{D}} = \alpha \vec{p}_{1,\text{un}}$, where the normalized (i.e. unscaled) singular vector $\vec{p}_{1,\text{un}}$ corresponds to the smallest singular value s_1 and the scaling coefficient α allows f to equal the input value. Here we use an input parameter, whereas in our experimental work,[2] we estimate a parameter.

The smallest singular value λ_1 will only equal zero exactly in the limit that the noise σ approaches zero, and λ_2 is larger. Nevertheless, λ_2 may approximate zero, which would correspond to a 2D solution space. If we interpret the solution space as 2D, then $D = 2$ known values is sufficient to define a unique solution $\hat{\vec{p}}_{2\text{D}} = \alpha \vec{p}_{1,\text{un}} + \beta \vec{p}_{2,\text{un}}$ from within the 2D space, and, while any two choices of parameters are options, here we will fix both f and m_1 to equal the input values and allow the other parameters to be scaled accordingly.

If we assume the solution space is 3D, we require $D = 3$ known values to define a unique solution $\hat{\vec{p}}_{3\text{D}} = \alpha \vec{p}_{1,\text{un}} + \beta \vec{p}_{2,\text{un}} + \gamma \vec{p}_{3,\text{un}}$; for these three known values, here we use f, m_1, and m_2 for the dimer or f, m, and k for the monomer, leaving the damping b as the only unknown parameter in the monomer case.

Ultimately, we obtain three possible solutions for the parameters, $\hat{\vec{p}}_{1\text{D}}, \hat{\vec{p}}_{2\text{D}}$, or $\hat{\vec{p}}_{3\text{D}}$, where the 1D solution is the easiest to obtain, requiring the least additional information. For an experimentalist, the choice of solution space dimension (the nullity D) may be informed by running simulations and by considering the size of the singular values. If the second smallest singular value λ_2 is low, then the 1D

solution space may be insufficient and a 2D solution space may be more accurate, as shown in **Fig. 4d**. In such a case, an experimentalist seeking to calculate the physical parameters using NetMAP has three choices: (1) assume a 2D solution space (or higher dimensional) and find a solution in that space, (2) use the 1D solution space solution, recognizing that the error will be larger, or (3) take additional spectral data to improve the accuracy of the 1D solution. In any of these situations, simulations may inform the choice.

In order to validate the accuracy of NetMAP, we use *a priori* information from the simulations (step 6 of **Fig. 1c**) to calculate the discrepancy $\Delta p_j = \hat{p}_j - p_{j,\text{in}}$ between the input and output parameters, where j indexes the parameters. The fractional discrepancy, $\frac{\Delta p_j}{p_{j_\text{in}}} = \frac{\hat{p}_j}{p_{j_\text{in}}} - 1$, has a normal distribution over multiple trials and is shown as box and whisker plots (**Fig. 2c** and **Fig. 3e**). The percent error for each parameter, the absolute value of the fractional discrepancy:

$$e_j = \frac{|\Delta p_j|}{p_{j_\text{in}}} \cdot 100\%, \tag{S10}$$

describes the accuracy of the output parameters. Because we take an absolute value, E_j has a half-normal distribution. When we repeat the simulated experiment multiple times with different random noise for each repetition, then we obtain a logarithmic average $\bar{E}_j$ over multiple trials of the error. We calculate the average error over parameters as

$$\langle e \rangle = \frac{e_1 + e_2 + \cdots + e_N}{N - D} \tag{S11}$$

where N is the total number of parameters and D is the number of parameters that have been fixed such that their error is zero, which should not contribute to the arithmetic mean. We consider the mean error $\overline{\langle e \rangle}$ averaging over parameters and multiple trials, to be the figure of merit that describes the accuracy of the SVD approach in describing a resonator system, with a lower error $\overline{\langle e \rangle}$ indicating a high accuracy. The input parameters are *a priori* information, only available when conducting simulations, and generally not available when conducting experiments, so we seek to predict the average error $\langle \bar{e} \rangle$ from values available to an experimentalist, including the correlation R-value and the signal to noise ratio (SNR). We compute the correlation R-value:

$$1 - R^2 = \frac{\text{SSres}}{\text{SStot}} \tag{S12}$$

to quantify the agreement between the input and output spectra (step 7 in **Fig. 1c**). To compute the R-value, we solve the equations of motion and calculate the output spectra $\hat{Z}_i(\omega)$ for each resonator i from the recovered parameters $\hat{\vec{p}}$, and plot both the noisy input spectra $\vec{Z}(\omega)$ (colorful datapoints) and the output spectra $\hat{\vec{Z}}(\omega)$ (black dashed line) for $n_R \approx 100$ frequencies, many more frequencies than we use for NetMAP in the examples here. Since we may plot either the amplitude and phase or the real and imaginary parts of the complex amplitude Z_i, we have a choice of which plots to use for

calculating the R-value. For a monomer, we choose both $\mathrm{Re}(Z)$ vs ω and $\mathrm{Im}(Z)$ vs ω, calculate R^2 for each and use the arithmetic mean as our value for R^2 in **Fig. 2d**. For a dimer, we choose all four of $\mathrm{Re}(Z_1)$ vs ω, $\mathrm{Re}(Z_2)$ vs ω, $\mathrm{Im}(Z_1)$ vs ω, $\mathrm{Im}(Z_2)$ vs ω, calculate R^2 for each and use the arithmetic mean as our value for R^2 in **Fig. 3g**. Since we compare parameters output from the SVD analysis and measured spectra, the R-values are accessible to experimentalists, whereas directly calculating the error $\overline{\langle e \rangle}$ required *a priori* knowledge of the parameters. By providing a prediction for the error $\overline{\langle e \rangle}$, we will enable an experimentalist to estimate the accuracy of the parameters $\hat{\vec{p}}$ output from the SVD analysis without needing to know *a priori* the true parameters $\vec{p}$.

2.6 Factorial methods

To understand how the percent error of the algebraic response vector characterization varies with factors of our system, we ran full, replicated two-level factorial experiments—e.g. 2^k experiments,[6] where $k = N$. The input factors of these experiments include spring constants, damping, mass, the applied force, the standard deviation of the input noise of simulated spectra, and the number of frequencies sampled. We varied these factors over an order of magnitude. For the monomer study, $k = 6$ parameters, and for the dimer study, $k = 8$. Factors and levels for monomers and dimers are shown in **Table S1** and **Table S2**, respectively. The response variables for this experiment included the average error for one-, two-, and three-dimensional null spaces. In the case of two- and three-dimensional null spaces, we scaled the singular vectors as described above (Section 2.5). We replicated monomer experiments 29 times, for a total of 30 full simulated experiments. We replicated dimer experiments 9 times, for a total of 10 full simulated experiments. The results of the simulations were analyzed using ANOVA factor screening methods, and the model assumptions were checked and verified for each. We analyzed the logarithm of the error $\overline{\langle e \rangle}$ because the raw data did not satisfy the equal variances assumption, and the logarithm of a half-normal distribution better approximates a normal distribution. The logarithm stabilized the variance appropriately. We used $\alpha = 0.05$ significance cutoff for effects and interactions in the reduced model.

Table S1 Factorial parameters for monomer investigation, $2^k r, k = 6, r = 30$

Factorial for monomer, $\mathbf{2^6}$, 29 repeats	$-$	$+$
number of frequencies, n	2	10
Mass $\boldsymbol{m}$ (kg)	1	10
Damping $\boldsymbol{b}$ (N s/m)	0.1	1
Stiffness $\boldsymbol{k}$ (N/m)	1	10
Force amplitude $\boldsymbol{f}$ (N)	1	10
standard deviation $\boldsymbol{\sigma}$ (meters)	5×10^{-5}	5×10^{-4}

Table S2 Factorial parameters for dimer investigation, $2^k r, k = 8, r = 10$

Factorial for dimer: $\mathbf{2^8}$, 9 repeats	$-$	$+$
mass $\boldsymbol{m_1}$ (kg)	1	10
mass $\boldsymbol{m_2}$ (kg)	1	10
damping $\boldsymbol{b_1}$ (N s/m)	0.1	1
damping $\boldsymbol{b_2}$ (N s/m)	0.1	1
stiffness $\boldsymbol{k_1}$(N/m)	1	10
stiffness $\boldsymbol{k_2}$ (N/m)	1	10
coupling stiffness $\boldsymbol{k_{12}}$ (N/m)	1	10
Force amplitude $\boldsymbol{f}$ (N)	1	10

2.7 Simplifying assumptions

There are several simplifying assumptions we make for this investigation regarding how measurements are made and what information is available to an experimenter. We assume as an approximation that the resonant system is not over-driven, heated, or subject to temperature fluctuations. We assume that masses, spring stiffnesses, and damping coefficients are constant, regardless of the driving frequency or force.

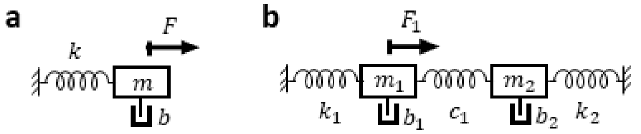

Figure S2 Cartoon of a) monomer and b) dimer system with two coupled masses.

We assume that the experimenter has a correct model for the number of resonant masses and springs and their connections, and that the system has reached a steady-state oscillation, where the energy dissipated by the damping balances the energy input by the force. For this investigation, we consider

the simplest two systems (**Figure S2**): a single damped driven resonant mass on a spring, which we call a monomer, and a resonant dimer system consisting of a pair of two masses, each connected to a wall by a spring and coupled together with a third spring.

For both the monomer and dimer presented here, we assume the experimenter drives one mass with a sinusoidal force $F = f\cos(\omega t)$ and measures the amplitude A and phase ϕ of each resonant mass, and the phase is measured with respect to the phase of the driving frequency.

The monomer system (**Figure S2a**) is fully described by the following parameters: mass m, spring stiffness k, damping coefficient b, and driving force $F(t)$. We assume that the driving force amplitude f and frequency ω are known. If, however, an experimenter does not know the driving amplitude f in Newtons, knowledge of any one of the other parameters, m, k, or b, is equivalently useful, or the experimenter can use NetMAP to obtain the force-normalized parameters, m/f, k/f, and b/f without requiring any additional information for a 1D solution space. In our related work,[2] we scale the parameters with respect to the spring constant, calculating m/k, b/k, and f/k. Higher dimensional solution spaces require more *a priori* information about the parameters.

The dimer system (**Figure S2b**) is fully described by the following parameters: masses m_1 and m_2, with damping coefficients b_1 and b_2, each attached to a wall by a spring of stiffnesses k_1 and k_2 and coupled to each other by a spring of stiffness c_1, also called k_{12}. We assume that the first mass of the dimer is driven and that there is no external driving force acting on the second mass, though our algebraic approach can be modified to address an additional driving force.

3. Monomers

3.1 Simulating a monomer

For a monomer (**Figure S2a**) driven by a sinusoidal force, the equation of motion in complex form is the differential equation

$$m\ddot{x} = f\mathrm{e}^{\mathrm{i}\omega t} - kx - b\dot{x} \tag{S13}$$

where x is the complex position of the mass, $\dot{x}$ is its complex velocity, $\ddot{x}$ is its complex acceleration, and the real part of the equation is the physical description. We guess a steady-state solution to this differential equation of the form $x = Z\,e^{\mathrm{i}\omega t}$, where Z is the complex amplitude of the position of the oscillating mass, and, through substitution for x and division by the oscillating factor $e^{i\omega t}$, obtain the time-independent equation of motion

$$(-\omega^2 m + \mathrm{i}\omega b + k)Z = f \tag{S14}$$

Separating into the real and imaginary parts, we find the equation of motions requires both

$$(-\omega^2 m + k)\,\mathrm{Re}(Z) - f = 0 \text{ and } -\omega b\,\mathrm{Im}(Z) = 0 \tag{S15}$$

We assume an experimenter would measure the complex amplitude $Z = Ae^{i\phi}$ of the single oscillator in response to the applied force $f e^{i\omega t}$.

3.2 Recovering monomer parameters using SVD

We next calculate the unknown parameters m, b, and k from the known values f, ω, and Z. Normally, the response $Z(\omega)$ is measured at a series of driving frequencies ω and fit with an iterative least-squares approach that optimizes $1 - R^2$, where R^2 is the coefficient of determination. We describe an alternative non-iterative approach using singular value decomposition. We require at least $n = 2$ measurements of the complex amplitude, $Z(\omega_1)$ and $Z(\omega_2)$, and with singular value decomposition (SVD), the method supports any number of measurements above two, as follows. For each measurement $Z(\omega_i)$, we know that both Eq 3a and 3b must hold, and we organize these equations as follows:

$$\mathcal{Z}\,\vec{p} = \vec{0}$$

$$\begin{bmatrix} -\omega_1^2\,\mathrm{Re}\big(Z(\omega_1)\big) & -\omega_1\mathrm{Im}\big(Z(\omega_1)\big) & \mathrm{Re}\big(Z(\omega_1)\big) & -1 \\ -\omega_1^2\,\mathrm{Im}\big(Z(\omega_1)\big) & \omega_1\mathrm{Re}\big(Z(\omega_1)\big) & \mathrm{Im}\big(Z(\omega_1)\big) & 0 \\ -\omega_2^2\,\mathrm{Re}\big(Z(\omega_2)\big) & -\omega_2\mathrm{Im}\big(Z(\omega_2)\big) & \mathrm{Re}\big(Z(\omega_2)\big) & -1 \\ -\omega_2^2\,\mathrm{Im}\big(Z(\omega_2)\big) & \omega_2\mathrm{Re}\big(Z(\omega_2)\big) & \mathrm{Im}\big(Z(\omega_2)\big) & 0 \end{bmatrix} \begin{bmatrix} m \\ b \\ k \\ f \end{bmatrix} = \begin{bmatrix} 0 \\ 0 \\ 0 \\ 0 \end{bmatrix} \tag{S16}$$

where the measurement matrix $\mathcal{Z}$ is calculated from the n spectrum measurements. For the monomer, each frequency contributes two rows to $\mathcal{Z}$, so $n = 2$ frequencies provide a 4×4 square matrix. We have flexibility in the number of measured frequencies by expanding to a rectangular matrix, where each additional frequency adds an additional two rows to the matrix. We assume an experimenter's measurement of each element of the measurement matrix $\mathcal{Z}$ is subject to Gaussian noise affecting Z but that the noise in frequency ω_i is negligible. In order to algebraically solve the differential equation, we apply singular value decomposition (SVD) to the matrix to obtain its singular values and corresponding 4-dimensional singular vectors. For a square normal matrix with a normal eigenbasis, we may also call these the eigenvalues and eigenvectors. The singular value corresponding to zero has a singular vector corresponding to the parameters vector $\vec{p}$. We use the known force amplitude f to normalize the parameters vector, thereby obtaining the physical parameters $\hat{m}$, $\hat{b}$, and $\hat{k}$, where the hat symbol indicates that these are calculated from our model of the measured data.

3.3 Details for the monomer case shown in Figure 2 of the main text

3.3.1 Scaling

Here we provide details about the 1D and 2D scaling for the monomer shown in **Fig. 2** of the main text. For this monomer, we set the input values to m = 4 kg, b = 0.01 N s/m, k = 16 N/m, and f = 1 N, so $\omega_{\mathrm{res}} \approx 1.99999922$ rad/s and we approximate the quality factor as $Q \sim \frac{\sqrt{mk}}{b} = 800$. We set the input noise to have a standard deviation of σ =0.005 m. We select 2 frequencies for SVD analysis, namely ω_a =1.99999922 rad/s and ω_b = 2.00125039 rad/s. These are circled in **Fig. 2ab**.

The matrix for SVD analysis is

$$\mathcal{Z} = \begin{bmatrix} -1.2629 \times 10^{-1} & 1.00004 \times 10^{2} & 3.1573 \times 10^{-2} & -1 \\ 2.00008 \times 10^{2} & 6.3147 \times 10^{2} & -5.0002 \times 10^{1} & 0 \\ 1.0007 \times 10^{2} & 5.0011 \times 10^{1} & -2.4985 \times 10^{1} & -1 \\ 1.0009 \times 10^{2} & -5.0002 \times 10^{1} & -2.4990 \times 10^{1} & 0 \end{bmatrix}.$$

The smallest singular value, $\lambda_1 = 1.0676 \times 10^{-7}$, corresponds to singular vector

$$\hat{\vec{p}} = (\hat{m}, \hat{b}, \hat{k}, f) = \alpha\,(-0.2421 \text{ kg}, -0.0006053 \text{ N/(m/s)}, -0.96836 \text{ N/m}, -0.060536 \text{ N}),$$

where

$$\alpha = f_{\mathrm{in}}/-0.060536 \text{ N} = 1 \text{ N}/-0.060536 \text{ N} = -16.519$$

is a scaling coefficient obtained from our knowledge of the force amplitude f for a 1D-SVD analysis. Dividing by α allows us to scale the singular vector to yield the modeled parameters vector. We thus obtain $\hat{m} = 3.99912$ kg, $\hat{b} = 0.0099996 \frac{\text{N}}{\text{m/s}}$, and $\hat{k} = 15.99647$ N/m. The percent errors for each of these is −0.022%, −0.0040%, and 0.022%, respectively. Each of these is within 0.022% of the correct values for m, b, and k. We also see that the recovered value

$$\sqrt{\frac{\hat{k}}{\hat{m}}} = 2.000000008 \text{ rad/s}$$

is more accurate than the individually recovered values for mass and spring stiffness. The percent error for $\sqrt{\frac{\hat{k}}{\hat{m}}}$ compared to $\sqrt{\frac{k_{\mathrm{in}}}{m_{\mathrm{in}}}}$ is 4.1×10^{-7}%. This high accuracy likely arises because we choose frequency ω_a at the peak amplitude and the lightly damped monomer has a sharply peaked resonance, so $\sqrt{k/m}$ is well defined.

3.3.2 2D Solution space for the monomer example

In our example, the vector

$$\beta\,\vec{p}_{2,\mathrm{un}} + \alpha\,\vec{p}_{1,\mathrm{un}} = \beta[\text{-1.62549828e-02, 9.98136999e-03, -5.83247471e-02, 9.98115410e-01}] + \alpha\,[\text{-2.42090968e-01, -6.05227330e-04, -9.68363873e-01, -6.05227821e-02}]$$

spans the 2D solution space, but we need to determine both α and β to obtain the physical parameters. For example, if we assume the experimenter independently knows both $f_{in} = 1$ N and $m_{\mathrm{in}} = 4$ kg, then it is straightforward to obtain the coefficients $\alpha = -16.5227$ and $\beta = -6.60069\text{e-}7$ to then obtain $\hat{b} = 0.009999992$ and $\hat{k} = 15.999999993$. Generating the curves from these two recovered parameters yields $R_A^2 = 1 - 1 \times 10^{-11}$ and $R_\phi^2 = 1 - 4 \times 10^{-7}$. In this case, the 1D solution and 2D solution were almost the same. We note that $\alpha \gg \beta$, thus selecting a 2D solution almost entirely in the 1D solution space. The 1D solution is preferred because it requires independent knowledge of only 1 parameter, and we find that it is often reasonably accurate, even when the 2D or 3D solution is more accurate.

3.4 Details for the monomer case shown in Figure 4 of the main text

For the monomer in **Figure 4**, the maximum number of frequencies is $n = 25$. We set the input values to: $m = 4$ kg, $b = 0.4 \frac{\mathrm{N}}{\mathrm{m/s}}$, $k = 10$ N/m, $f = 1$ N. Then the resonance frequency $\omega_{\mathrm{res}} = 1.58$ rad/s and

$$Q = \frac{\sqrt{mk}}{b} = 15.8.$$

We set the input noise $\sigma = 0.0005$ m. We use up to 25 frequencies for SVD analysis, namely [1.5795569 1.5961569 1.5629569 1.6127569 1.5463569 1.6293569 1.5297569 1.6459569 1.5131569 1.6625569 1.4965569 1.6791569 1.4799569 1.6957569 1.4633569 1.7123569 1.4467569 1.7289569 1.4301569 1.7455569 1.4135569 1.7621569 1.3969569 1.7787569 1.3803569] rad/s.

The spectrum with these 25 frequencies identified is shown in **Figure S3, left**. For $n = 25$ frequencies, the $\boldsymbol{Z}$ matrix has 50 rows. Its smallest singular value, $\lambda_1 = 0.00059708$, corresponds to singular vector $\hat{\vec{p}} = (\hat{m}, \hat{b}, \hat{k}, f)$ =α(−0.36955 kg, −0.036957 N/(m/s), −0.92387 N/m, −0.092383 N), where α=f_{in}/ −0.092383 N = 1 / −0.092383 = −10.825 is a normalization constant obtained from our knowledge of the force amplitude f for a 1D-SVD analysis. Dividing by α allows us to scale the singular vector to yield the modeled parameters vector. Therefore, we obtain $\hat{m} = 4.00017$ kg, $\hat{b} = 0.400043$ N/(m/s) and $\hat{k}$ = 10.00047 N/m. The percent errors for each of these is 0.0041835%, 0.010689%, and 0.0046745%, respectively. Each of these is within 0.010689% of the correct values for m, b, and k. We also see that the recovered value

$$\sqrt{\frac{\hat{k}}{\hat{m}}} = 1.5811 \text{ rad/s}$$

is more accurate than the individually recovered values for mass and spring stiffness; this is often true for various sharply peaked systems. The percent error for $\sqrt{\frac{\hat{k}}{\hat{m}}}$ compared to $\sqrt{\frac{k_{\text{in}}}{m_{\text{in}}}}$ is 0.00025%. Heatmaps in **Figure 4c** of the main text show how accurately $n = 2$, 1D-SVD and 2D-SVD recover the solution for this monomer. **Figure S3, right** shows a similar heatmap for $n = 2$, 3D-SVD. For a monomer, 3D-SVD only recovers damping b; all other parameters must be known *a priori* in order to find the solution in the 3D solution space, so the average error is showing the error in damping $\bar{e}_b$ (**Figure S3, right**).

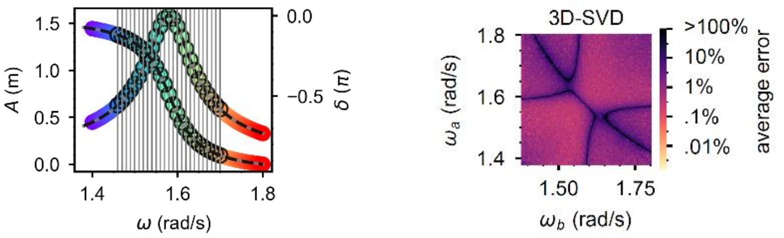

Figure S3. (left) The spectrum also shown in **Fig. 4a**, with corresponding color scale. Vertical lines and circles show the $n = 25$ frequencies used for analysis. δ is the phase. (right) Heatmap showing the average error for $D = 3, n = 2$ analysis of the same monomer system analyzed in Figure 4.

Figure S4 shows how the average error for the monomer parameters decreases for 1D-SVD as the number of frequency points increases. A connecting letters report (**Table S3**) shows that there are diminishing returns to increasing the number of frequency points: $n = 25$ is not significantly better than $n = 7$. **Figure S5** expands on **Fig. 4f**, showing the variation of the error for 1D-SVD for both of the smallest two singular values. Since the error varies more with the second smallest singular value λ_2 than with the smallest singular value λ_1, **Figure 4f** in the main text shows the variation only with λ_2.

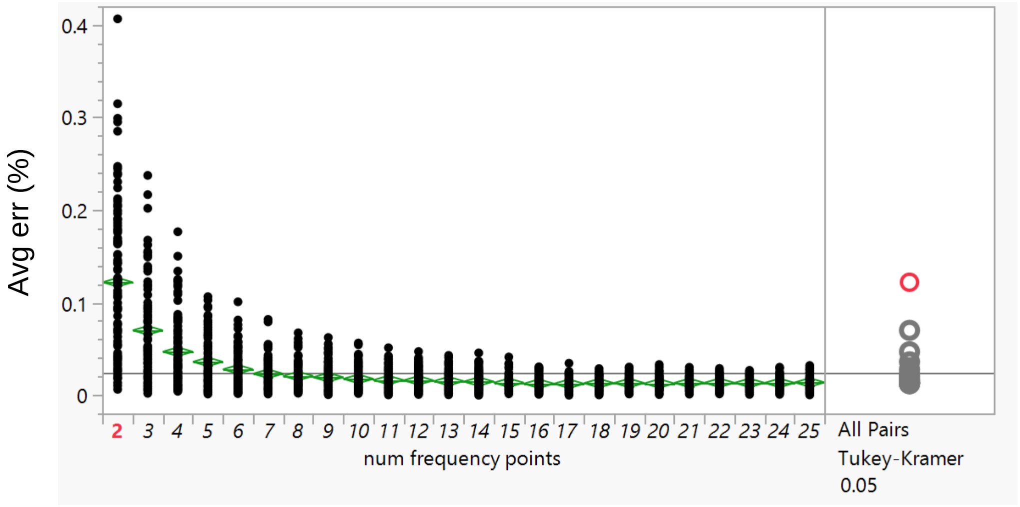

Figure S4. Average error $\langle e \rangle$ for m, k, b as a function of the number of frequency points used in the SVD analysis of a monomer. These are the same datapoints as **Fig. 4b** for 1D-SVD in the main text.

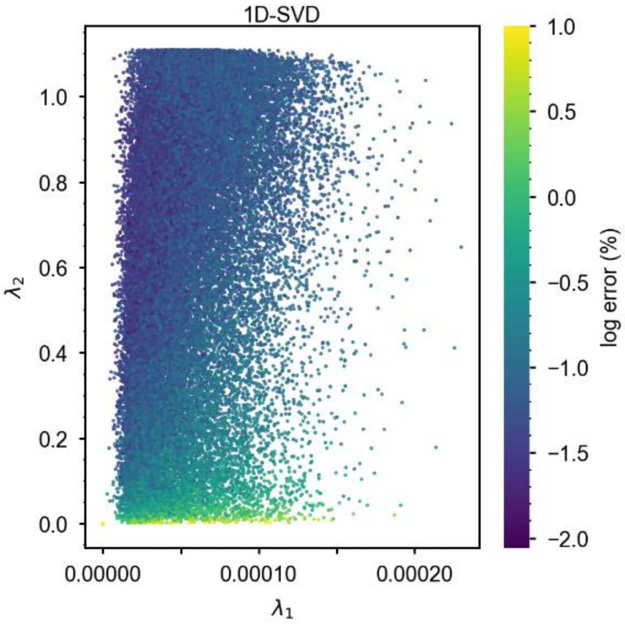

Figure S5. The 1D solution is much less accurate when λ_2 is small. For λ_2 near zero, the error of the 1D solution rises to 10%, and for larger λ_2, the 1D solution error is as low as 0.01%. This is the same dataset shown in **Fig. 4f** of the main text.

Table S3. Connecting letters report for the data shown in **Figure S4**, corresponding to **Fig. 4b**.

n	connecting letters						Avg err (%)
2	A						0.12234
3		B					0.07037
4			C				0.04724
5			C	D			0.03635
6				D	E		0.02823
7					E	F	0.02347
8					E	F	0.02103
9					E	F	0.01964
10					E	F	0.01792
11					E	F	0.01660
12					E	F	0.01628
13						F	0.01543
14						F	0.01497
15						F	0.01373
16						F	0.01273
17						F	0.01266
18						F	0.01329
19						F	0.01345
20						F	0.01298
21						F	0.01356
22						F	0.01367
23						F	0.01361
24						F	0.01357
25						F	0.01406

Levels not connected by same letter are significantly different.

4. Dimers

4.1 Simulating the dimer

We consider how the algebraic approach presented here applies to a dimer system of damped driven oscillators: two masses m_1 and m_2, with damping b_1 and b_2, respectively, each coupled to a wall by a spring of stiffness k_1 and k_2, respectively, and coupled to each other with a spring of stiffness k_{12} (**Figure S2b**). The equations of motion of the damped driven dimer in complex form are the coupled differential equations

$$m_1\ddot{x}_1 = f_1 e^{\mathrm{i}\omega t} - b_1\dot{x}_1 - k_1 x_1 - k_{12}(x_1 - x_2) \text{ and}$$

$$m_2\ddot{x}_2 = f_2 e^{\mathrm{i}\omega t} - b_2\dot{x}_2 - k_2 x_2 - k_{12}(x_2 - x_1), \quad \text{(S17)}$$

where f_1 is the amplitude of the driving force applied to the first oscillator and f_2 is the amplitude of the oscillating driving force applied to the second oscillator. The two forces are assumed to have the same frequency and phase, such that the force amplitudes f_1 and f_2 are each real constants, or one of

the two is assumed to be zero. We guess a steady-state solution of the form $x_1 = Z_1 e^{\mathrm{i}\omega t}$, $x_2 = Z_2 e^{\mathrm{i}\omega t}$, where $Z_1 = A_1 e^{\mathrm{i}\phi_1}$ and $Z_2 = A_2 e^{\mathrm{i}\phi_2}$ are the complex amplitudes of the respective oscillating masses. Then the equations of motion (B1) give us

$$(-m_1\omega^2 + k_1 + k_{12} + \mathrm{i}\omega b_1)Z_1 - k_{12}Z_2 = f_1 \text{ and}$$
$$-k_{12}Z_1 + (-m_2\omega^2 + k_{12} + k_2 + \mathrm{i}\omega b_2)Z_2 = f_2. \tag{S18}$$

In order to simulate the spectra, we organize these two equations into a matrix equation,

$$\begin{bmatrix} -m_1\omega^2 + k_1 + k_{12} + \mathrm{i}\omega b_1 & -k_{12} \\ -k_{12} & -m_2\omega^2 + k_{12} + k_2 + \mathrm{i}\omega b_2 \end{bmatrix} \begin{bmatrix} Z_1 \\ Z_2 \end{bmatrix} = \begin{bmatrix} f_1 \\ f_2 \end{bmatrix} \tag{S19}$$

and use Cramer's rule to calculate the spectra $z_1(\omega)$ and $z_2(\omega)$ for a given set of physical parameters $\vec{p}_{\mathrm{in}} = (m_1, m_2, b_1, b_2, k_1, k_2, k_{12}, f_1, f_2)$. As before, we add random Gaussian noise $\Gamma_{x,i}(\sigma,\omega) + \mathrm{i}\Gamma_{y,i}(\sigma,\omega)$ to the spectra to simulate noisy experimental conditions.

4.2 Recovering the dimer parameters with SVD

Having now simulated an experiment, we consider how we can recover the physical parameters from the noisy spectra $Z_1(\omega)$ and $Z_2(\omega)$ and knowledge of the driving forces $F_1 = f_1 e^{\mathrm{i}\omega t}$ and $F_2 = f_2 e^{\mathrm{i}\omega t}$. We reorganize Eq (B2) into a different matrix equation than we used for Cramer's rule by creating a vector $\vec{p}$ of parameters and a matrix $\mathcal{Z}$ of measurements such that:

$$\mathcal{Z}\,\vec{p} = \vec{0}$$

$$\begin{bmatrix}
-\omega_1^2 X_{11} & 0 & -\omega_1 Y_{11} & 0 & X_{11} & 0 & X_{11} - X_{21} & -1 & 0 \\
-\omega_1^2 Y_{11} & 0 & \omega_1 X_{11} & 0 & Y_{11} & 0 & Y_{11} - Y_{21} & 0 & 0 \\
0 & -\omega_1^2 X_{21} & 0 & -\omega_1 Y_{21} & 0 & X_{21} & X_{21} - X_{11} & 0 & -1 \\
0 & -\omega_1^2 Y_{21} & 0 & \omega_1 X_{21} & 0 & Y_{21} & Y_{21} - Y_{11} & 0 & 0 \\
\vdots & \vdots & \vdots & \vdots & \vdots & \vdots & \vdots & \vdots & \vdots \\
-\omega_n^2 X_{1n} & 0 & -\omega_n Y_{1n} & 0 & X_{1n} & 0 & X_{1n} - X_{2n} & -1 & 0 \\
-\omega_n Y_{1n} & 0 & \omega_n X_{1n} & 0 & Y_{1n} & 0 & Y_{1n} - Y_{2n} & 0 & 0 \\
0 & -\omega_n^2 X_{2n} & 0 & -\omega_n Y_{2n} & 0 & X_{2n} & X_{2n} - X_{1n} & 0 & -1 \\
0 & -\omega_n^2 Y_{2n} & 0 & \omega_n X_{2n} & 0 & Y_{2n} & Y_{2n} - Y_{1n} & 0 & 0
\end{bmatrix}
\begin{bmatrix} m_1 \\ m_2 \\ b_1 \\ b_2 \\ k_1 \\ k_2 \\ k_{12} \\ f_1 \\ f_2 \end{bmatrix}
=
\begin{bmatrix} 0 \\ 0 \\ 0 \\ 0 \\ 0 \\ 0 \\ 0 \\ 0 \\ 0 \end{bmatrix}$$

where $X_{ji} = \mathrm{Re}\left(Z_j(\omega_i)\right)$ and $Y_{ji} = \mathrm{Im}\left(Z_j(\omega_i)\right)$ provide information about measurements of each resonator at each of the discrete frequency points.

The measurement matrix $\mathcal{Z}$ has $\dim(\vec{p})$=9 columns and $4n$ rows for the dimer, where n is the number of frequency points used for the analysis. In general we have N_{cluster} complex equations describing the system. Taking the real and imaginary parts, we rewrite these as $2N_{\mathrm{cluster}}$ real equations and thus

the real measurement matrix $\mathcal{Z}$ has $2nN_{\text{cluster}}$ rows. For example, using measurements from two frequencies to describe a dimer would require an 8×9 matrix whereas using 30 frequencies would require a 120×9 matrix $\mathcal{Z}$. We then analyze the measurement matrix $\mathcal{Z}$ using SVD to identify the singular vectors and identify 1 or more singular vectors corresponding to the singular value of zero. The dimension of the solution space may be open to interpretation because the measurements are noisy, so the smallest singular values do not precisely equal zero. If we interpret the solution space to be 1-dimensional then there is one singular vector corresponding to the null singular value. We therefore scale the singular vector using our knowledge of one element of the parameters vector $\vec{p}$. In particular, here we assume we know force amplitude f_1 pushing the first mass. Hence the analysis recovers all but one of the elements of the parameters vector $\vec{p} = (m_1, m_2, b_1, b_2, k_1, k_2, k_{12}, f_1, f_2)$. The resulting output parameters $\hat{p}_i$ are not exactly equal to the input parameters $p_{i,\text{in}}$ due to noise in the measurements. If we do not add noise, the recovered parameters are exactly equal to the input parameters, within computer precision. Therefore, we add noise in order to validate NetMAP with noise.

4.3 Statistical comparison tests for dimer case

Table S4 shows the statistical comparison tests for the dimer case shown in **Figure 3** of the main text.

Table S4. Observed t-statistics and associated p-values for network parameters of the dimer shown in **Fig. 3** of the main text.

**Null-space Dimension**	$\hat{p}_j$	$t_0 \equiv \frac{\lvert(p_{\text{in}})_j - \hat{p}_j\rvert}{s.e._j}$	***p*-value**
**1D**	$\hat{m}_1$	1.8867	0.0595
**1D**	$\hat{b}_1$	0.3242	0.7458
**1D**	$\hat{k}_1$	1.7826	0.0750
**1D**	$\hat{m}_2$	1.2347	0.2172
**1D**	$\hat{b}_2$	0.4928	0.6223
**1D**	$\hat{k}_2$	1.2431	0.2141
**1D**	$\hat{k}_{12}$	1.9109	0.0563
**2D**	$\hat{b}_1$	0.9078	0.3642
**2D**	$\hat{k}_1$	0.9317	0.0750
**2D**	$\hat{m}_2$	1.1388	0.2551
**2D**	$\hat{b}_2$	0.7423	0.4581
**2D**	$\hat{k}_2$	1.1114	0.2667
**2D**	$\hat{k}_{12}$	0.1713	0.8640

4.4 Error is inversely proportional to the SNR

Since the error is proportional to the input noise σ, the error is also inversely proportional to the SNR, as shown in **Figure S6**.

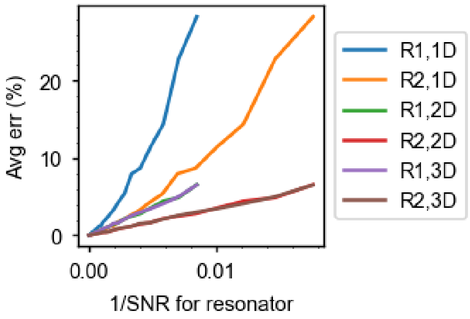

Figure S6. For the dimer shown in **Fig. 3** of the main text, error is inversely proportional to the SNR. This is another view of the data shown in **Fig. 3h** of the main text, where input noise is varied. The average SNR for resonator 1 (R1) and resonator 2 (R2) also varies, and the error of the recovered parameters is proportional to the input noise. The lines here are noisy but linear.

4.5 Details for the dimer case shown in Figure 5 of the main text

Here we provide details relating to the dimer case shown in **Figure 5** of the main text. **Figure S7** shows the R1 and R2 spectra for this dimer. The second resonance peak in (a), near 3.501 rad/s, is imperceptible on the amplitude spectrum but does show an effect in the phase spectrum. **Figure 5c** of the main text shows how the accuracy of the 1D solution improves as additional response vectors are incorporated into the analysis. The improvement shows diminishing benefits as the number of response vectors becomes large. A connecting letters report (see **Table S5**) shows which of the errors are significantly different from each other. Levels sharing a letter are not significantly different from each other.

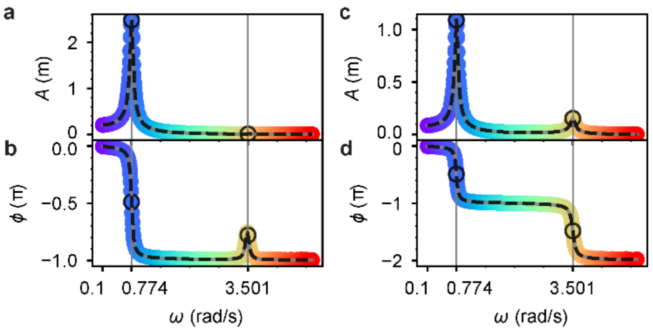

Figure S7. Spectra corresponding to Fig. 5 of the main text. **a)** Amplitude $A_1(\omega)$, **b)** Phase $\phi_1(\omega)$, **c)** Amplitude $A_2(\omega)$, **d)** Phase $\phi_2(\omega)$. The black circles and vertical grey lines indicate the two input frequencies $\omega_a = 0.774$ rad/s and $\omega_b = 3.501$ rad/s, each at a resonant peak, used for the SVD analysis ($n = 2$. The subtle grey curves show the exact spectra calculated from input parameters using Cramer's rule. The colorful datapoints show simulated spectra with added noise $\sigma = 5 \times 10^{-5}$ m. The dashed black line shows the output spectra $\hat{A}$ and $\hat{\phi}$ calculated from the 1D-SVD recovered parameters $\hat{\vec{p}}$ for one trial, showing agreement with the exact spectra in grey and with the noisy simulated spectra in rainbow colors.

Table S5. Connecting letters report for the 1D solutions shown in **Fig. 5c** of the main text.

n	Connecting letters														Avg err (%)
2	A														0.50053
3	A														0.48953
4		B													0.38672
5		B	C												0.37370
6		B	C	D											0.31921
7			C	D	E										0.31322
8				D	E	F									0.27451
9				D	E	F	G								0.26418
10					E	F	G	H							0.24554
11						F	G	H	I						0.24172
12						F	G	H	I	J					0.22021
13						F	G	H	I	J	K				0.21480
14							G	H	I	J	K	L			0.19807
15								H	I	J	K	L	M		0.19438
16								H	I	J	K	L	M	N	0.19215
17								H	I	J	K	L	M	N	0.18881
18								H	I	J	K	L	M	N	0.18362
19								H	I	J	K	L	M	N	0.18024
20									I	J	K	L	M	N	0.17379
21										J	K	L	M	N	0.17000
22										J	K	L	M	N	0.16381
23										J	K	L	M	N	0.16226
24										J	K	L	M	N	0.15636
25										J	K	L	M	N	0.15441
26										J	K	L	M	N	0.15141
27											K	L	M	N	0.15042
28											K	L	M	N	0.14758
29											K	L	M	N	0.14581
30												L	M	N	0.14003
31												L	M	N	0.13893
32												L	M	N	0.13846
33												L	M	N	0.13749
34												L	M	N	0.13644
35												L	M	N	0.13581
36												L	M	N	0.13500
37												L	M	N	0.13462
38												L	M	N	0.13143
39												L	M	N	0.13085
40												L	M	N	0.13187
41												L	M	N	0.13145
42												L	M	N	0.13282
43												L	M	N	0.13217
44												L	M	N	0.13148
45												L	M	N	0.13103
46												L	M	N	0.12903
47												L	M	N	0.12864
48													M	N	0.12781
49													M	N	0.12709
50														N	0.12471

5. Experimental considerations for measuring absolute phase

Measuring the absolute phase requires some experimental consideration because the time elapsing between the driving force and the response usually is measured by a lock-in amplifier, and the electronics and optics will introduce delays before the driving force arrives at the resonator system and additional delays before the measurement can be read, creating a phase delay $\theta = \omega t_{\text{delay}}$, which is added onto the absolute phase ϕ. We assume that this time delay can be measured and subtracted to obtain an absolute phase, as follows[2]. An experimenter may obtain the time delay by measuring the spectra at lower frequencies, where the force and response are expected to be in phase such that the absolute phase ϕ is zero. Then the total phase $\theta + \phi = \omega t_{\text{delay}} + 0$, such that a linear fit of the phase versus frequency provides the slope t_{delay}. The phase delay θ is subtracted for each frequency ω to obtain the absolute phase ϕ.

6. Additional Cases

6.1 Heavily damped monomer

As an additional example, we consider a heavily damped monomer (step 1). We simulate a spectrum for a mass-and-spring where we set $m = 4$ kg, $b = 8$ N/(m/s), $k = 9$ N/m, $f = 1$ N, and Gaussian noise with standard deviation $\sigma = 0.0005$ m (step 2). With these input parameters, the quality factor is $Q \approx \frac{\sqrt{km}}{b} = 0.75$ and the resonant frequency is

$$\omega_r = \sqrt{\frac{k}{m} - \frac{b^2}{2m^2}} = 0.5 \text{ rad/s}.$$

(The approximate equation $\sqrt{k/m} = 1.5$ rad/s greatly overestimates the resonant frequency for the heavily damped case.) We mimic an experiment by simulating the spectrum with noise. We can use any frequency points for the analysis with SVD, and to demonstrate this we choose $n = 3$ frequencies at random, ω_1 =0.183389 rad/s, ω_2 =0.545455 rad/s, and $\omega_2 =$ 1.481318 rad/s, as shown in **Figure S8**. At these three measurement frequencies, we obtain amplitude $A(\omega_1)$ =0.111738 m, $A(\omega_2) =$ 0.112556 m, $A(\omega_3)$ =0.083927 m and phase $\phi(\omega_1) = -0.155537$ rad, $\phi(\omega_2) = -\ 0.507897$ rad, and $\phi(\omega_3) = -1.542800$ rad (step 3). The signal to noise ratio for each measurement is $\frac{A(\omega_1)}{\sigma}$ =223, $\frac{A(\omega_2)}{\sigma} = 224$ and $\frac{A(\omega_3)}{\sigma} = 169$, and the mean SNR is 205. We use a parameters vector $\vec{p} = (m, k, b, f)$ and thus construct the real rectangular 6×4 matrix (step 4)

$$\mathcal{Z} = \begin{bmatrix} -\omega_1^2\,\mathrm{Re}(Z(\omega_1)) & -\omega_1\mathrm{Im}(Z(\omega_1)) & \mathrm{Re}(Z(\omega_1)) & -1 \\ -\omega_1^2\,\mathrm{Im}(Z(\omega_1)) & \omega_1\mathrm{Re}(Z(\omega_1)) & \mathrm{Im}(Z(\omega_1)) & 0 \\ -\omega_2^2\,\mathrm{Re}(Z(\omega_2)) & -\omega_2\mathrm{Im}(Z(\omega_2)) & \mathrm{Re}(Z(\omega_2)) & -1 \\ -\omega_2^2\,\mathrm{Im}(Z(\omega_2)) & \omega_2\mathrm{Re}(Z(\omega_2)) & \mathrm{Im}(Z(\omega_2)) & 0 \\ -\omega_3^2\,\mathrm{Re}(Z(\omega_3)) & -\omega_3\mathrm{Im}(Z(\omega_3)) & \mathrm{Re}(Z(\omega_3)) & -1 \\ -\omega_3^2\,\mathrm{Im}(Z(\omega_3)) & \omega_3\mathrm{Re}(Z(\omega_3)) & \mathrm{Im}(Z(\omega_3)) & 0 \end{bmatrix} \tag{S20}$$

and obtain the singular vector (step 5)

$$\hat{\vec{p}}_{1D} = (\hat{m},\, \hat{b},\, \hat{k},\, f) = \alpha\left(-0.21709\ \mathrm{kg}, -0.50854\frac{\mathrm{N}}{\mathrm{m/s}}, -0.83167\frac{\mathrm{N}}{\mathrm{m}}, -0.05080\ \mathrm{N}\right),$$

which we scale with the known input force amplitude $f = 1$ N such that $\alpha = -0.05080$ to obtain

$$(\hat{m},\, \hat{b},\, \hat{k}, f) = \left(4.2734\ \mathrm{kg}, 10.010\frac{\mathrm{N}}{\mathrm{m/s}}, 16.371\frac{\mathrm{N}}{\mathrm{m}}, 1\mathrm{N}\right).$$

Comparing $\vec{p}_{\mathrm{in}}$ and $\hat{\vec{p}}$, the error for each parameter is $e_m = 6.8\%$, $e_b = 0.10\%$, and $e_k = 2.3\%$, for an average of $\langle e \rangle = 3.1\%$ (step 6).

Calculating the resonance frequency from the recovered parameters is not very accurate when the resonance frequency is so broadened by the heavy damping. Whereas the lightly damped monomer in the main text has high accuracy for recovering $\sqrt{\frac{\hat{k}}{\hat{m}}}$, for this heavily damped monomer, the calculation is less accurate. We have $\sqrt{\frac{\hat{k}}{\hat{m}}} = 1.9573$ rad/s with error $e_{\sqrt{k/m}} = 30\%$, and

$$\sqrt{\frac{\hat{k}}{\hat{m}} - \frac{\hat{b}^2}{2\hat{m}^2}} = 1.0427\ \mathrm{rad/s}$$

with error 109%. These errors for estimated resonance frequency are much higher than the error for the individual parameters, suggesting that we do not have correlation errors for this case.

Hence a hypothetical experimenter would obtain $\hat{m}$, $\hat{b}$, and $\hat{k}$ with an average error e of 3.1% using just three randomly selected measurements of Z with an average signal to noise ratio of 205.

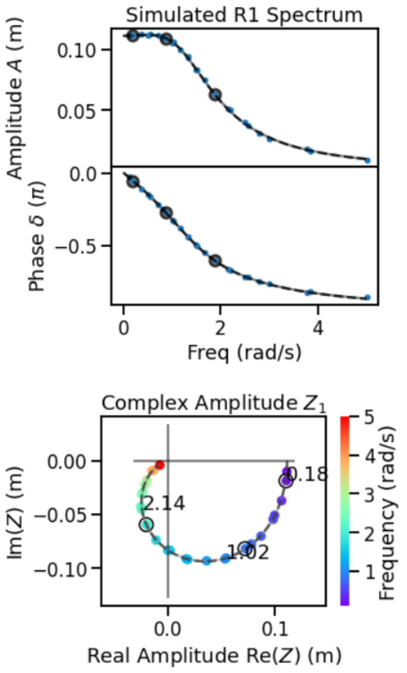

Figure S8. An example of a heavily damped monomer and 1D-SVD results. In each plot, a subtle grey curve shows the simulated spectrum $z(\omega)$ without noise. Colorful datapoints show the simulated noisy spectrum $Z(\omega) = Ae^{i\delta}$, with color indicating frequency. The three circled points in each plot represent the three randomly selected frequencies that are used for analysis. The black dashed curves show the recovered spectrum $\hat{Z}(\omega)$.

6.2 Medium-coupled dimer

As an additional example, we consider a dimer with medium coupling, and consider applying an oscillating force to resonator 1 (R1). We set the input values to $m_1 = 11$ kg, $b_1 = 0.5$ N s/m, $k_1 = 9$ N/m, f_1 = 1 N, $m_2 = 5$ kg, $b_2 = 0.1$ N s/m, $k_2 = 20$ N/m, and $k_{12} = 4$. The quality factors are

$$Q_1 \approx \frac{\sqrt{m_1 k_1}}{b_1} = 20 \quad \text{and} \quad Q_2 \approx \frac{\sqrt{m_2 k_2}}{b_2} = 100.$$

We set the input noise to σ =0.0005 m. We measure at the two resonance frequencies, 1.05 and 2.21 rad/s. Then the mean SNR for resonator 1 measurements is 193 and for resonator 2 measurements is 83.3. The spectra, histogram, and error versus noise are shown in Figure S9. For this dimer system, we find that the 3D solution is slightly more accurate than the 1D solution, and the 2D solution is significantly less accurate.

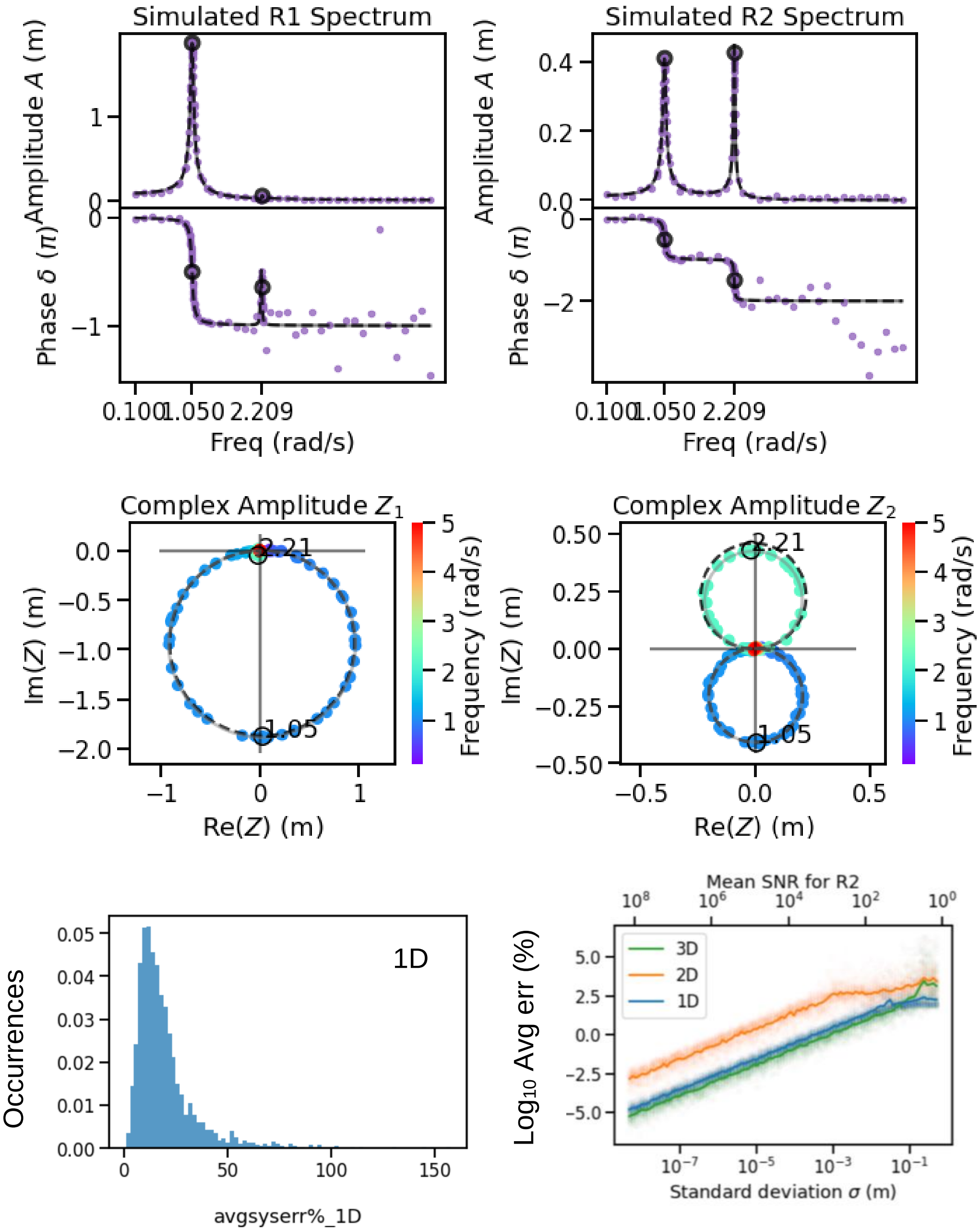

Figure S9. A medium-damped dimer. Resonator 1 has one strong resonance peak and one weak resonance peak, while the two resonance peaks for resonator 2 are equally strong and appear as two loops on the complex plane. The two peak frequencies (circled) are identified for SVD analysis. The 1D-SVD output spectra $\hat{\vec{Z}}(\omega)$ are shown as dashed black lines, and we see that the amplitude of the 2.2 rad/s peak for resonator 2 is not perfectly recovered. The histogram shows the distribution of $\langle e \rangle$ for 1D-SVD.

6.3 Strongly coupled dimer

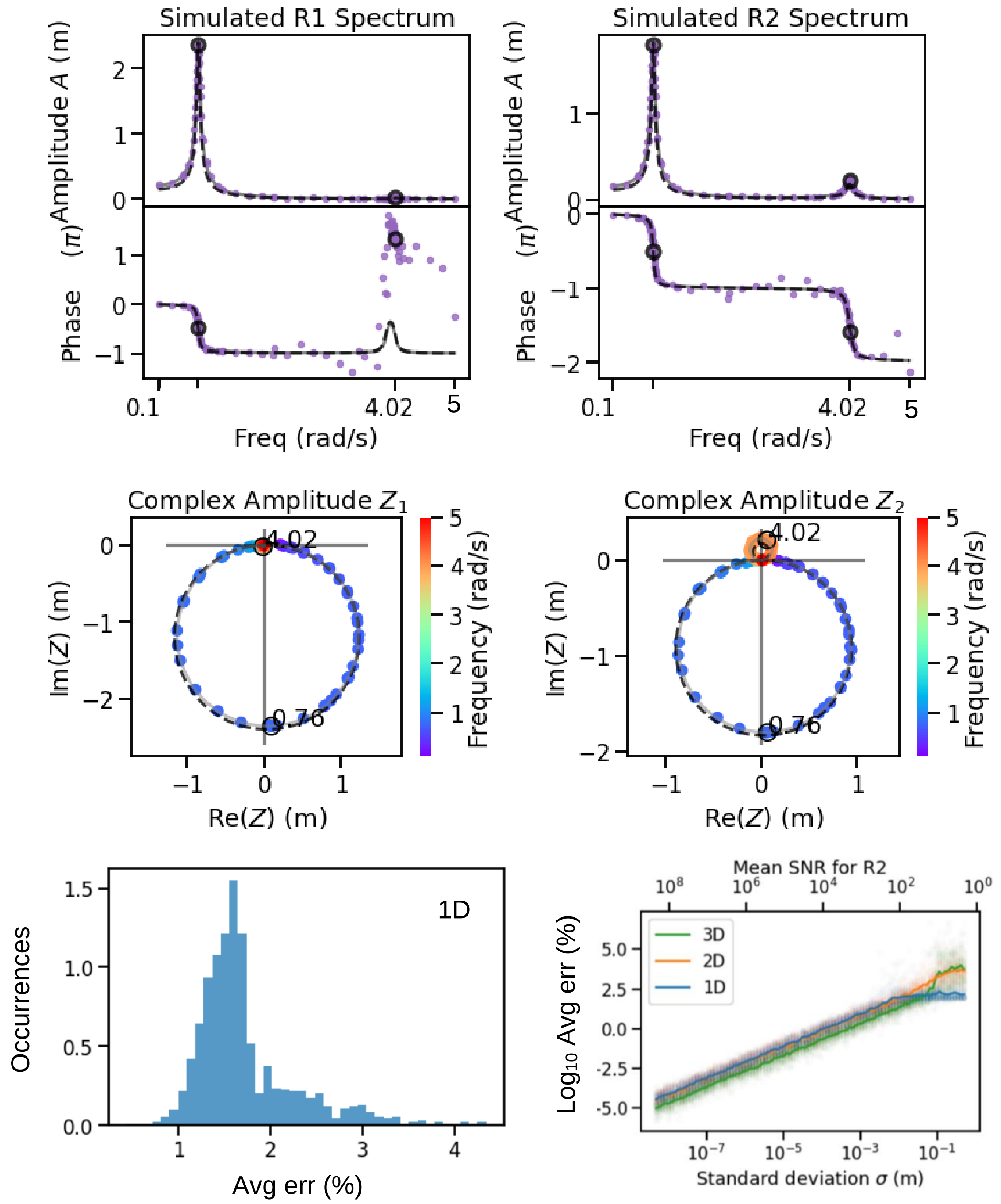

Figure S10. An example with a strongly coupled dimer. The spectra and histogram show a 1D solution.

As an additional example, we consider a dimer with strong coupling, and consider applying an oscillating force to resonator 1 (R1). We set the input values to $m_1 = 8$ kg, $b_1 = 0.5$ N s/m, $k_1 = 2$ N/m, $f_1 = 1$ N, $m_2 = 1$ kg, $b_2 = 0.1$ N s/m, $k_2 = 4$ N/m, and $k_{12} = 11$ N/m. We set the input noise to $\sigma = 0.0025$ m. We measure at the two resonance frequencies, 0.758 and 4.02 rad/s. Then the mean SNR for resonator 1 measurements is 239 and for resonator 2 measurements is 202. For the strongly coupled dimer shown in **Figure S10**, we find that the 3D solution is the most accurate, while the 1D and 2D solutions are similar to each other.

6.4 Force applied to both resonators of a dimer

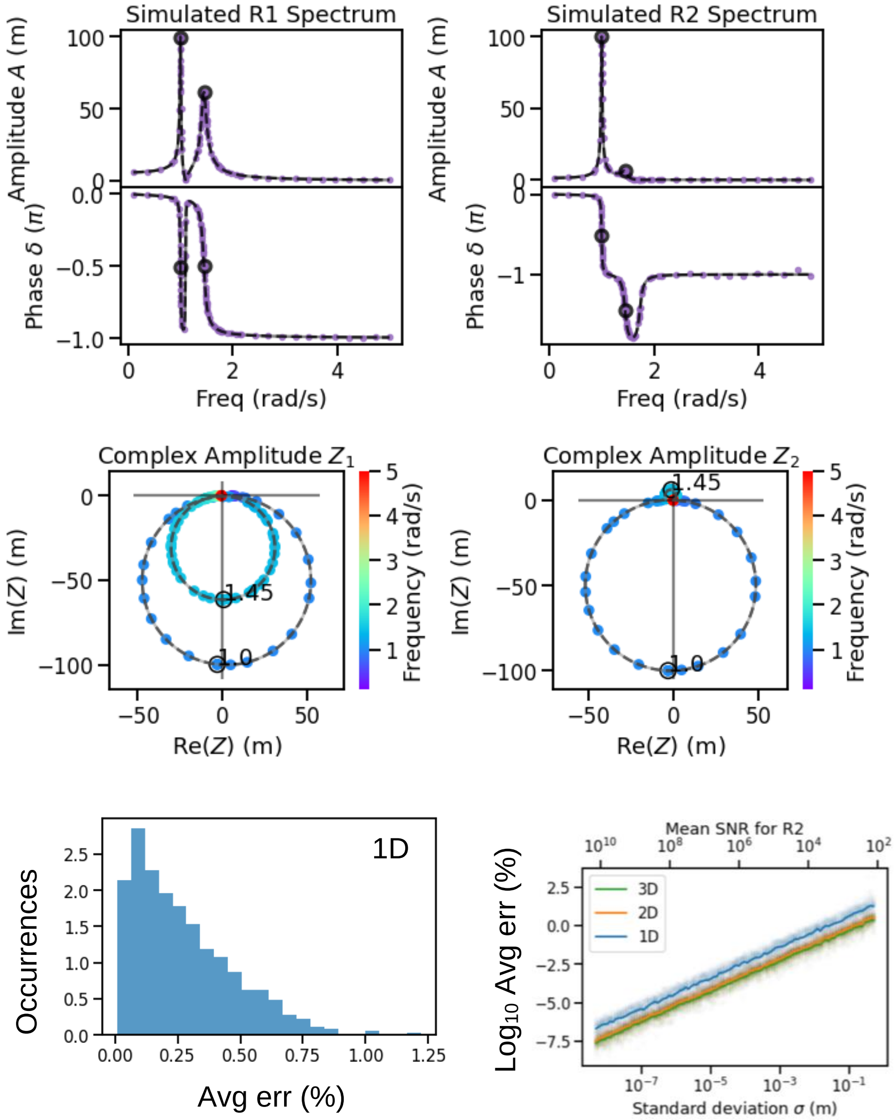

Figure S11. An example of a dimer where the same oscillating force is applied to both resonators

As an additional example, we consider applying a force $\vec{F} = \langle f \cos \omega t\,, f \cos \omega t \rangle$ to a dimer. That is, applying the same oscillating force to both masses. We set the input values to: $m_1 = 1$ kg, $b_1 = 0.1$ N s/m, $k_1 = 1$ N/m, $f_1 = f_2 = 10$ N, $m_2 = 10$ kg, $b_2 = 0.1$ N s/m, $k_2 = 10$ N/m, $k_{12} = 1$ N/m, $\sigma = 0.005$ m, and we measure at the resonance frequencies, 1.00 and 1.45 rad/s. Then the mean SNR for resonator 1 measurements is 16130 and for resonator 2 measurements is 10630. The results are shown in **Figure S11**.

For the dimer system in **Figure S11** with force applied to both resonators, we find that the 3D solution is slightly more accurate than the 2D solution, and the 1D solution is the least accurate. The amplitude A_2 of the second resonator is higher because it is also driven, and therefore the signal to noise ratio for the second resonator is better than cases where only one of the two resonators is driven. This explains why the solutions are overall more accurate than for dimer cases with only one driven oscillator.

7. Factorial experiments varying every parameter between two levels

Plots summarizing the factorial experiments are provided here for monomers (**Figure S12**) and dimers (**Figure S13** and **Figure S14**).

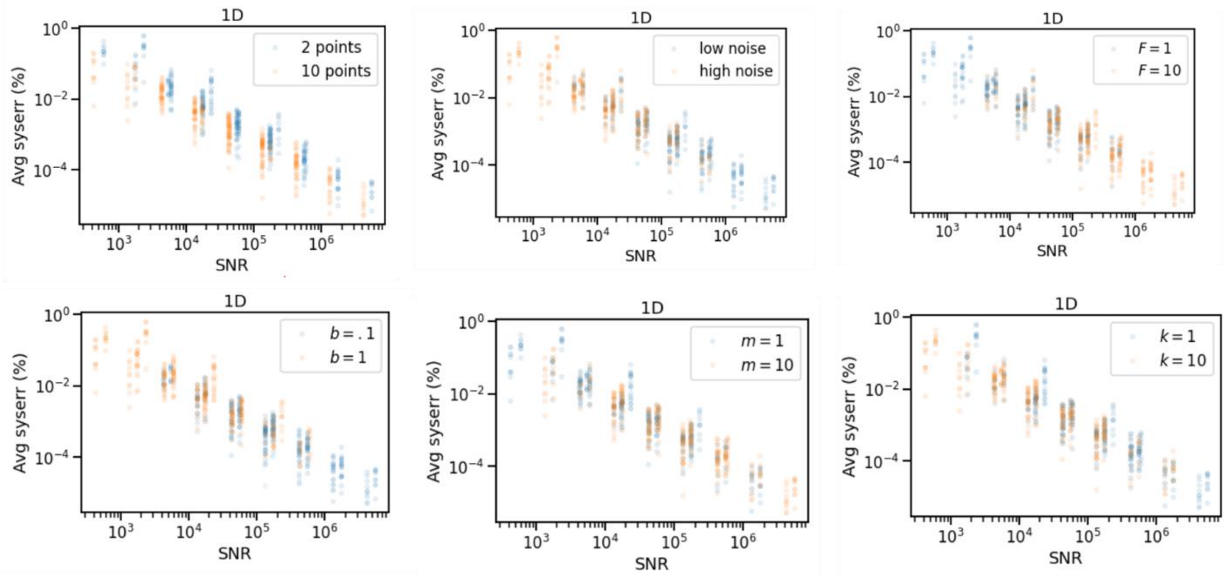

Figure S12. The 1D-SVD error for a monomer is inversely proportional to the SNR, and varies with n, m, and b. Each plot shows the complete set of data from the $2^k r$ experiments for the monomer described in **Table S1**. The color coding in each varies to show how the relationship between average SNR and average error varies with each of the six varied parameters, $2^k r, k = 6, r = 30$.

An experimentalist using NetMAP will wish to know the accuracy of the parameters vector without knowing the input parameters. A simplified linear model using R^2 and mean SNR (**Figure S13**) can estimate the average error for a wide range of situations.

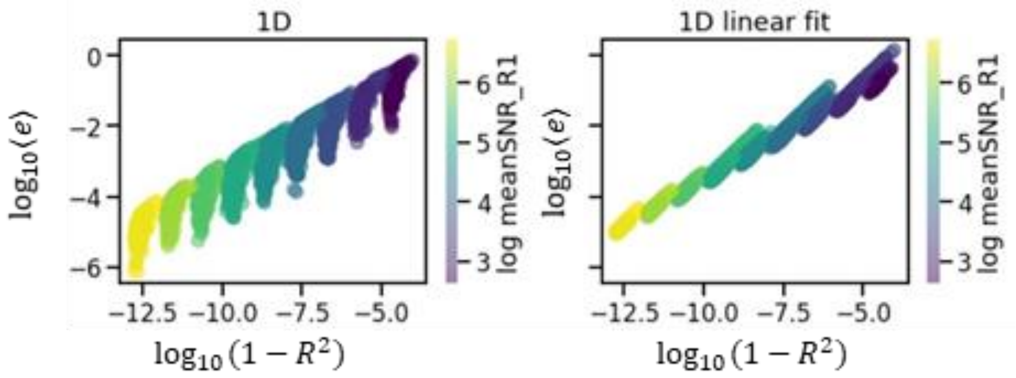

Figure S13. The results of the factorial simulations for dimers are shown on the left, showing that the error $\langle e\rangle$ (vertical axis) varies with $1 - R^2$ (horizontal axis) and SNR (color scale). On the right, a linear fit to the simulated data offers a simplified model for estimating the error from the SNR and R^2 values.

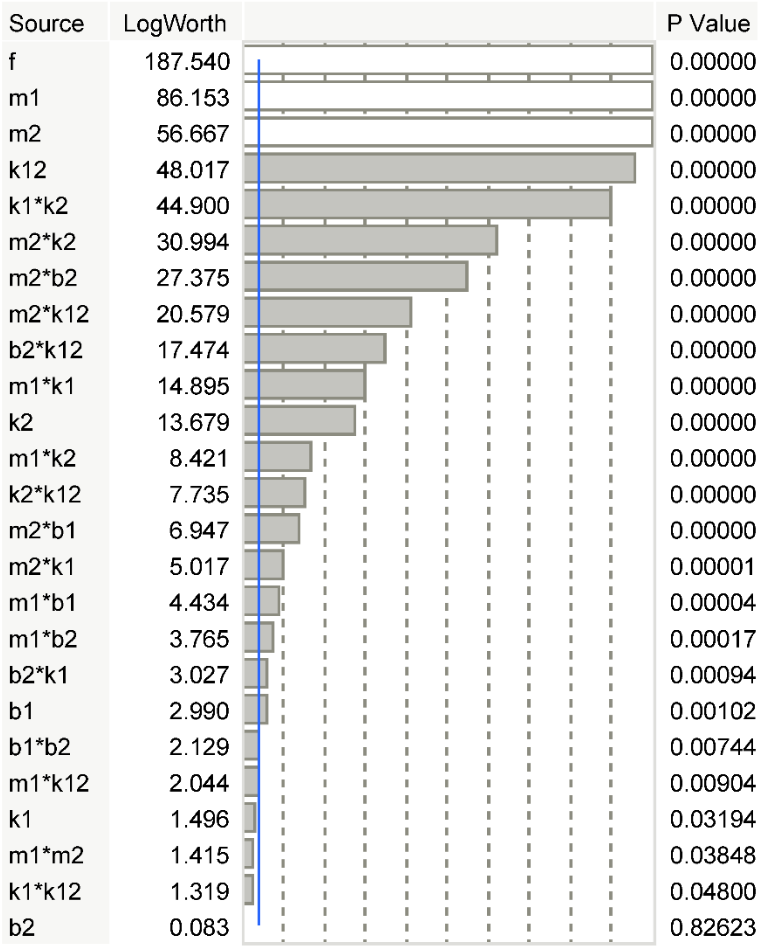

Figure S14. Reduced model effects summary of factorial experiment on the dimer system. The stronger effects and interactions have larger LogWorth values. Plots were generated in JMP software v 16.1.

1. Smith, C. *et al.* sympy/sympy: SymPy 1.11.1. (2022) doi:10.5281/zenodo.7035912.
2. Carter, B. *et al.* Spatial mapping and analysis of graphene nanomechanical resonator networks. Preprint at https://doi.org/10.48550/arXiv.2302.03680 (2023).
3. Miller, D. & Alemán, B. Spatially resolved optical excitation of mechanical modes in graphene NEMS. *Appl. Phys. Lett.* **115**, 193102 (2019).
4. Drmač, Z. & Veselić, K. New Fast and Accurate Jacobi SVD Algorithm. I. *SIAM J. Matrix Anal. Appl.* **29**, 1322–1342 (2008).
5. Drmač, Z. & Veselić, K. New Fast and Accurate Jacobi SVD Algorithm. II. *SIAM J. Matrix Anal. Appl.* **29**, 1343–1362 (2008).
6. Montgomery, D. C. *Design and Analysis of Experiments*. (John Wiley & Sons, 2008).
